# Pictorial key to species of the genus *Ropalidia* Guérin-Méneville, 1831 (Hymenoptera, Vespidae) from China, with description of one new species

**DOI:** 10.3897/zookeys.391.6606

**Published:** 2014-03-19

**Authors:** Jiang-Li Tan, Kees Van Achterberg, Xue-Xin Chen

**Affiliations:** 1 Key Laboratory of Resource Biology and Biotechnology in Western China (Northwest University), Ministry of Education; School of life Sciences, Northwest University, 229 North Taibai Road, Xi’an, Shaanxi 710069, China; 2 Naturalis Biodiversity Center, Dept. of Terrestrial Zoology, Postbox 9517, 2300 RA Leiden, the Netherlands; 3 Institute of Insect Sciences, Zhejiang University, Zijingang Campus, Yuhangtang Road 866, Hangzhou 310058, China

**Keywords:** Vespidae, Polistinae, *Ropalidia*, new records, new taxa

## Abstract

Twenty two species of the paper wasp genus *Ropalidia* Guérin-Méneville, 1831, are listed from China. Among them, *R. malaisei* van der Vecht, 1962, *R. cyathiformis* (Fabricius, 1804), *R. santoshae* Das & Gupta, 1989, *R. scitula* (Bingham, 1897), *R. obscura* Gusenleitner, 1996 and *R. ornaticeps* (Cameron, 1900) are new records from China. A new species, *R. parartifex* Tan & van Achterberg, is described. Their diagnostic characteristics are summarized in an illustrated key and 36 colourplates.

## Introduction

The genus *Ropalidia* Guérin-Méneville, 1831 (Hymenoptera: Vespidae: Polistinae: Ropalidiini), is the only polistine genus that includes both independent– and swarm-founding species, so that their nests are highly variable: arboreal or in cavities, envelope present or not (Carpenter and Nguyen 2003, Wenzel 1998). The genus can be separated from the other genera of the Ropalidiini by having the pretegular carina and dorsal episternal groove absent, the metasoma bell-shaped with its first metasomal segment petiolate and the second segment covering more or less the following segments which telescope one by one (Carpenter and Nguyen 2003). It is one of the largest polistine genera with more than 180 species and is distributed in the greater part of the Old World with a tropical or subtropical climate. The distribution is centered in the Oriental region, extending westward via Yemen to the Afrotropical region and southward to the Australian region (Kojima and Carpenter 1997, Saito and Kojima 2005a, b, Kojima 2006, Blommers 2012). The faunas of continental Africa, Australia, and New Guinea have a large number of endemic species with 18, 24 and 40 *Ropalidia* species, respectively. Most extreme is the fauna of Madagascar: all 43 species are endemic to Madagascar (Carpenter and Madl 2009, Blommers 2012). Both the Indian subcontinent (26 species) and South-East Asia (about 60 species) are very speciose (Kojima 2006, Nguyen et al. 2006). Although several taxonomic studies of the genus exist, the Chinese fauna of *Ropalidia* is still very poorly studied (Bingham 1897, van der Vecht 1941, 1962, Cheesman 1952, Richards 1978, Das and Gupta 1989, Giordani Soika 1991, Kojima 1982, 1984, 1996a, b, 1999a, b, Kojima and Tano 1985, Kojima et al. 2002, 2005, 2007, Nguyen et al. 2006). To date, 13 valid species and subspecies of *Ropalidia* have been reported from China, based mainly on research and collections in areas other than continental China (Sonan 1935, van der Vecht 1941, 1962, Das and Gupta 1989, Kojima et al. 2007, 2011, Nguyen et al. 2006, Kojima 2006). Liu (1936–1937) included 3 species of *Ropalidia* in his catalogue of the Vespidae of China; Lee (1982, 1985) recorded 10 species and subspecies with an obsolete key which only distinguished species by their colour. During the following 30 years, no comprehensive and complete taxonomic studies on this large fauna have been made. During 2012–2013, the first author studied the taxonomy of Chinese Vespidae in Leiden (RMNH), Paris (MNHN) and Hangzhou (ZJUH).The result is a new illustrated key to 22 species of the genus *Ropalidia* from China. Six species are recorded from China for the first time and one new species is described. Distributional data are extracted from the literature as well as based on specimens examined during this study; three colour plates are added to enhance the chance of a correct identification.

## Material and methods

Some specimens were collected with interception traps (Li et al. 2012, Sheng et al. 2013), but most specimens were collected with a hand net. The examined specimens are preserved in the Zoological Collection of School of Life Sciences, Northwest University, Xi’an (NWUM); the Parasitic Hymenoptera Collection of Institute of Insect Sciences, Zhejiang University, Hangzhou (ZJUH); the General Station of Forest Pest Management, State Forestry Administration, Shenyang (GSFA), the Entomological Museum of Northwest A&F University, Yangling (NWAM); the Environment and Plant Protection Research Institute, Chinese Academy of Tropical Agriculture Sciences, Haikou (CATAS); the Taiwan Agriculture Research Institute, Taichung (TARI); the Naturalis Biodiversity Center, Leiden (RMNH); the Natural History Museum, London (BMNH); the Museum National d’Histoire Naturelle, Paris (MNHN); the Zoological Museum of the University of Copenhagen (ZMUC); the Senckenberg Deutsches Entomologisches Institut, Müncheberg (SDEI) and the Oberösterreichisches Landesmuseum (Linz).

For the morphological and micro-sculpture terminology used in this paper see Kojima (1999c), Kojima et al. (2002, 2007) and Nguyen et al. (2006). An Olympus SZX 12 binocular microscope with analySIS Soft Imaging System software was used for the descriptions, measurements and photos.

### Key to species of the genus *Ropalidia* from China

**Table:** 

1	Propodeum with pair of raised vertical carinae anteriorly (a); propodeal orifice narrow, slit-like, acute above (b)	2
	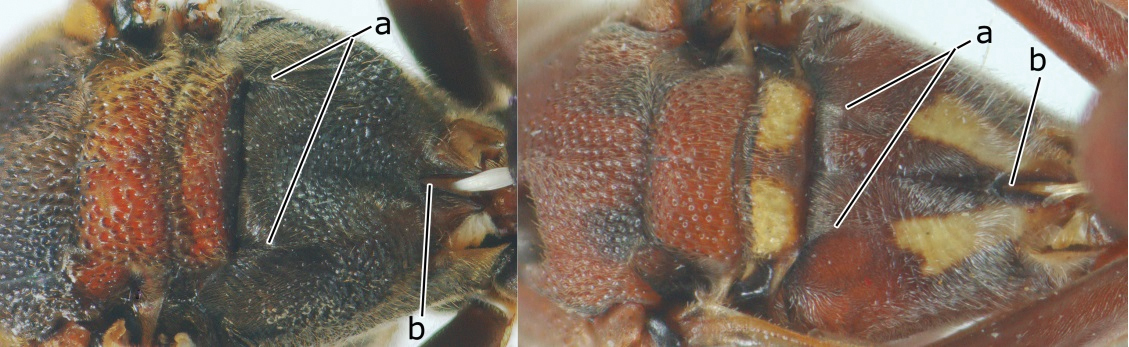	
–	Propodeum without pair of raised vertical carinae anteriorly (aa); propodeal orifice relatively broad, more or less rounded above (bb)	3
	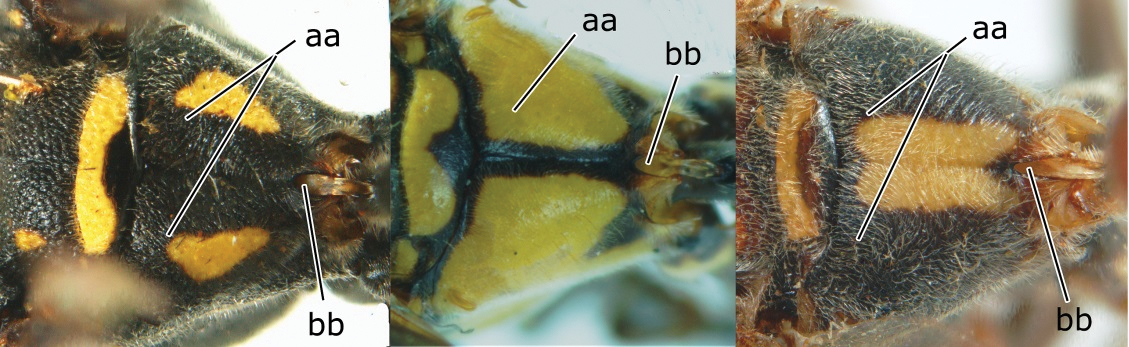	
2	Head narrower than mesoscutum just in front of tegulae (a); clypeus slightly higher than wide, shiny and its dorsal half impunctate (b); apical margin of second metasomal tergite armed with a row of spines (c)	*Ropalidia binghami* van der Vecht, 1941
	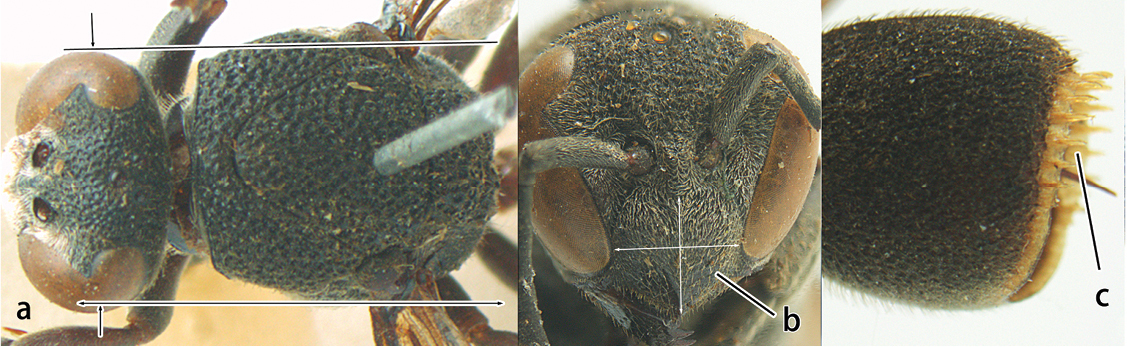	
–	Head wider than mesoscutum just in front of tegulae (aa); clypeus distinctly wider than high, dull and its dorsal half evenly punctate (bb); apical margin of second tergite simple (cc)	*Ropalidia marginata* (Lepeletier, 1804)
	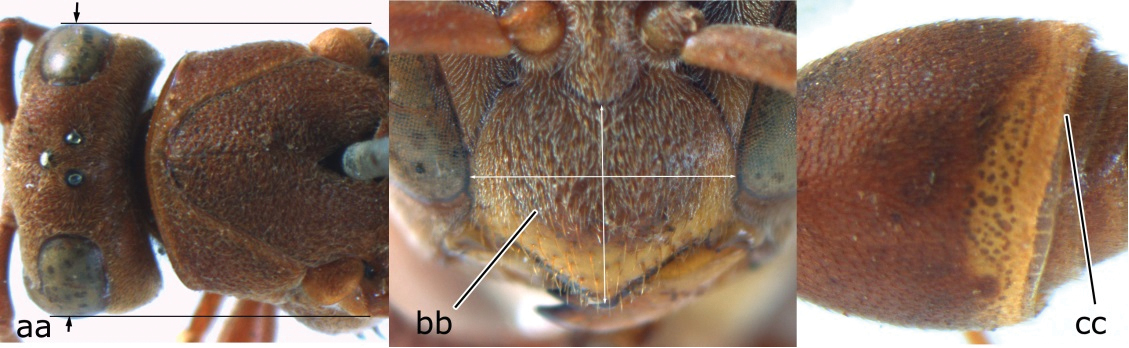	
3	Propodeal valvula large, obscuring most of propodeal orifice in lateral view (a); basal angle of second submarginal cell less than (b) or equal to or slightly greater than 90°(b’)	4
	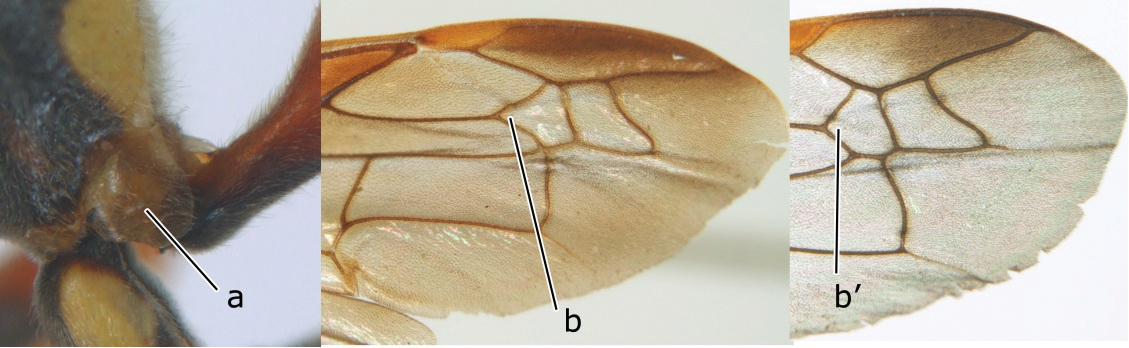	
–	Propodeal valvula medium-sized, most of propodeal orifice visible in lateral view (aa); basal angle of second submarginal cell greater than 90°(bb)	14
	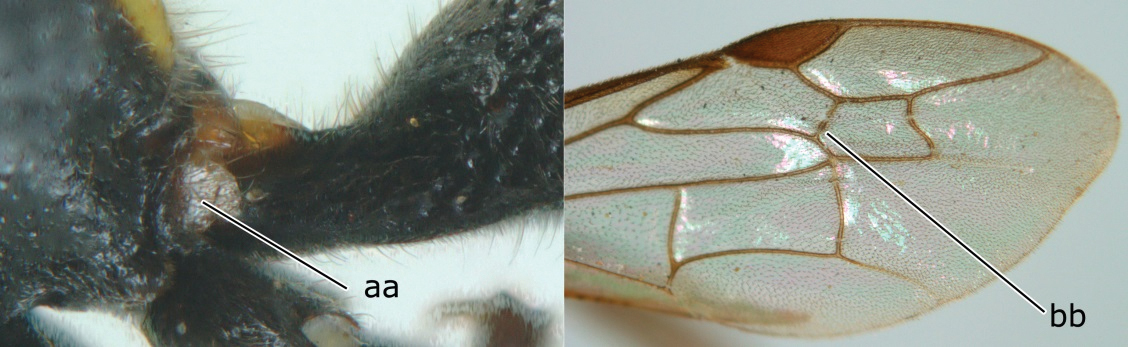	
4	Length between ocellus and eye (OOL) less than twice as long as length between posterior ocelli (POL) (a); first flagellomere of female antenna comparatively short, less than 2.5× as long as its apical width (b); basal angle of second submarginal cell ≥ 90°(c)	5
	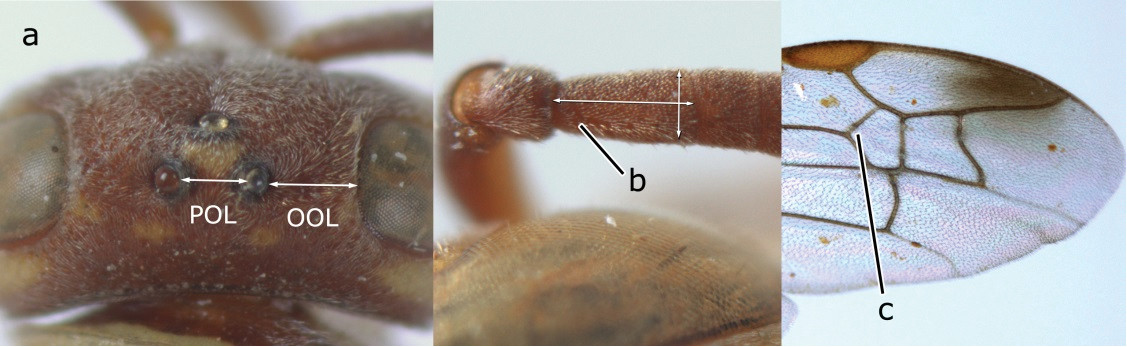	
–	OOL more than 2.5 × as long as POL (aa); first flagellomere of female antenna long, 3 × longer than its apical width (bb); basal angle of second submarginal cell distinctly less than 90°(cc)	6
	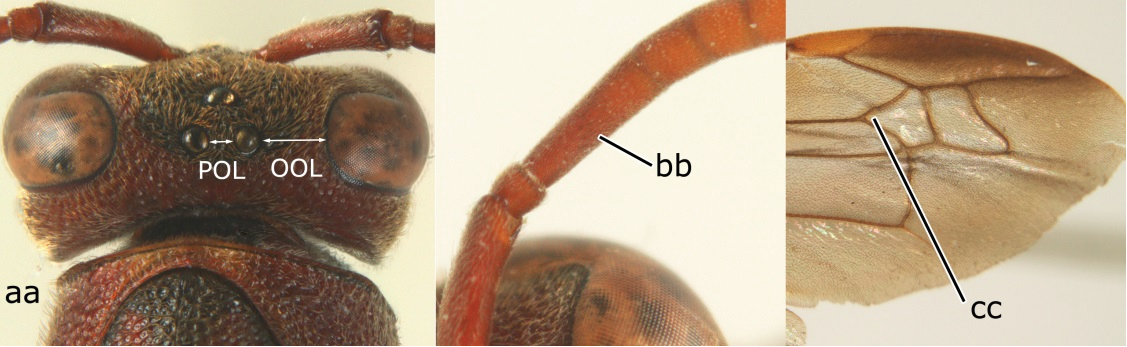	
5	Pronotal carina nearly straight (a); propodeum distinctly obliquely striate (b); propodeal valvula large, nearly circular (c); first metasomal tergite comparatively long, with petiolus parallel-sided, widened part swollen submedially and narrowed near apical margin (d); second metasomal segment oblique apically, with tergite longer than sternite (e); male antenna with distinct tyloids, apical two thirds of apical flagellomere excavated and curved (f)	*Ropalidia fasciata* (Fabricius, 1804)
	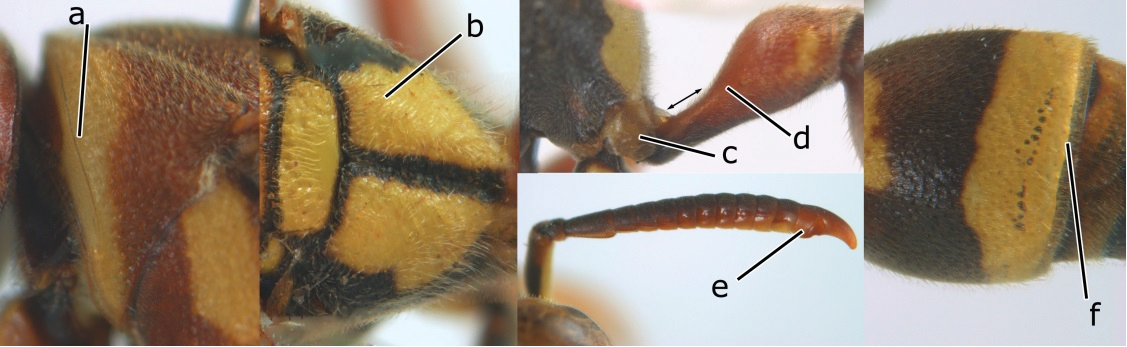	
–	Pronotal carina distinctly sinuate (aa); propodeum largely smooth (bb); propodeal valvula rounded triangular (cc); first tergite comparatively short, petiolus widened basally and apically, and widened part swollen up to apical margin (dd); second metasomal segment vertically cut off apically, with tergite about as long as sternite (ee); male antenna without tyloids and apical flagellomere normal (ff)	*Ropalidia variegata* (Smith, 1852)
	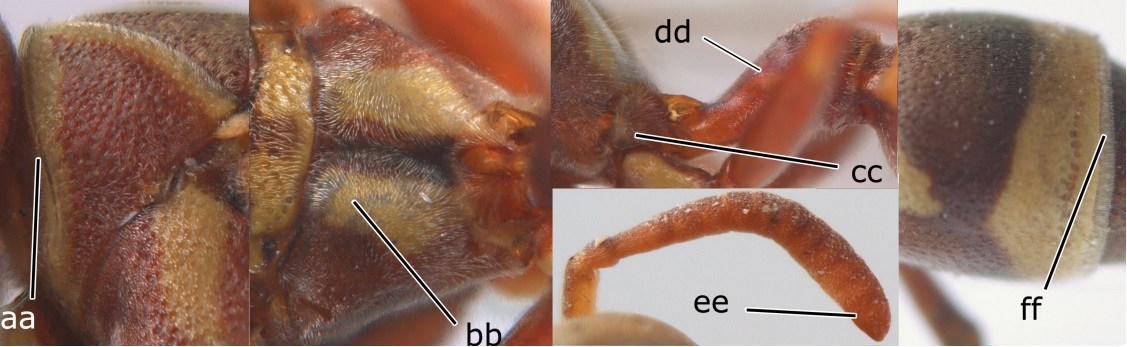	
6	Propodeum reticulate-punctate, anteriorly with distinct median depression (a), yellow marks of propodeum absent (b), dorsal side of propodeum reticulate-punctate (c); first metasomal tergite less than twice as long as wide in dorsal view and less than 2.5× as long as high in lateral view (d)	7
	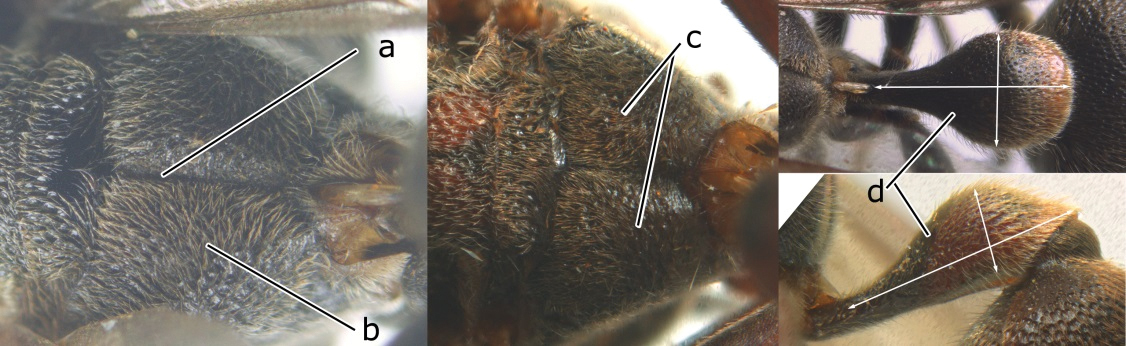	
–	Propodeum finely striate, barely depressed antero-medially or if depressed, only as fine median furrow (aa); propodeum with yellow marks confluent basally (bb); dorsal side of propodeum barely punctate, only finely transversely striate (cc); first tergite more than twice as long as wide in dorsal view and more than 2.5× as long as high in lateral view (dd)	9
	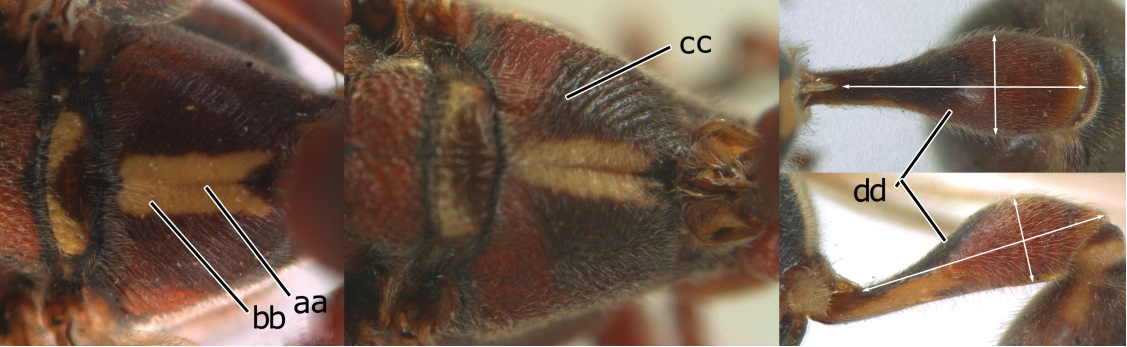	
7	Second metasomal tergite as wide as long in dorsal view (a); second segment longer than high in lateral view (b); scutellum black, slightly convex and without median furrow (c)	*Ropalidia santoshae* Das & Gupta, 1989, rec. n.
	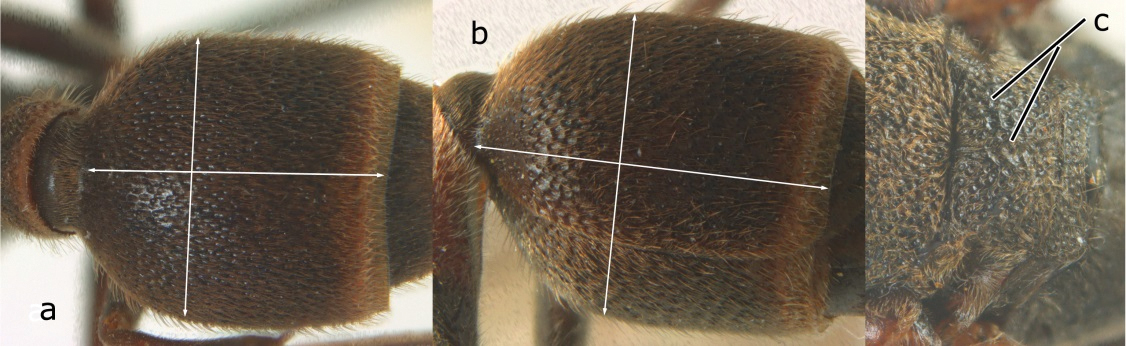	
–	Second tergite wider than long in dorsal view (aa); second segment as long as high in lateral view (bb); scutellum reddish brown, distinctly convex and with a longitudinal median furrow (cc)	8
	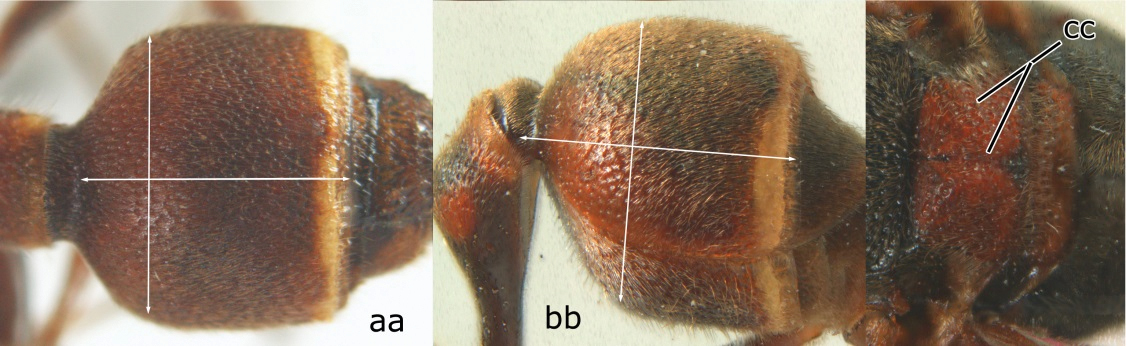	
8	Apical flagellomere of male antenna long; carina of the 8^th^ flagellomere (VIII) emarginate (a); second metasomal segment oblique apically, with sternite longer than tergite (b). Male: proximo-ventral margin of penis with a short hook, at most one fourth as long as penis valve (c)	*Ropalidia taiwana* Sonan, 1935
	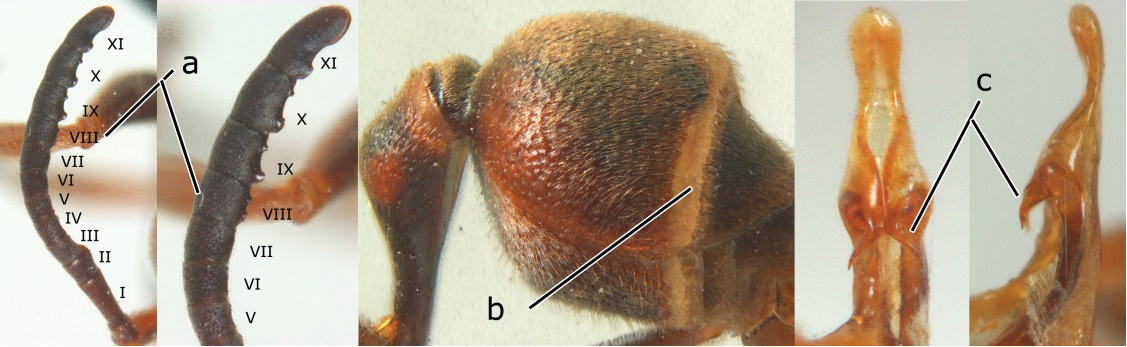	
–	Apical flagellomere of male antenna short; carina of the 8^th^ flagellomere (VIII) not emarginate (aa); second metasomal segment vertical apically, with tergite about as long as sternite (bb); male genitalia: proximo-ventral margin of penis valve with a long hook, two thirds as long as penis valve (cc)	*Ropalidia birmanica* van der Vecht, 1962
	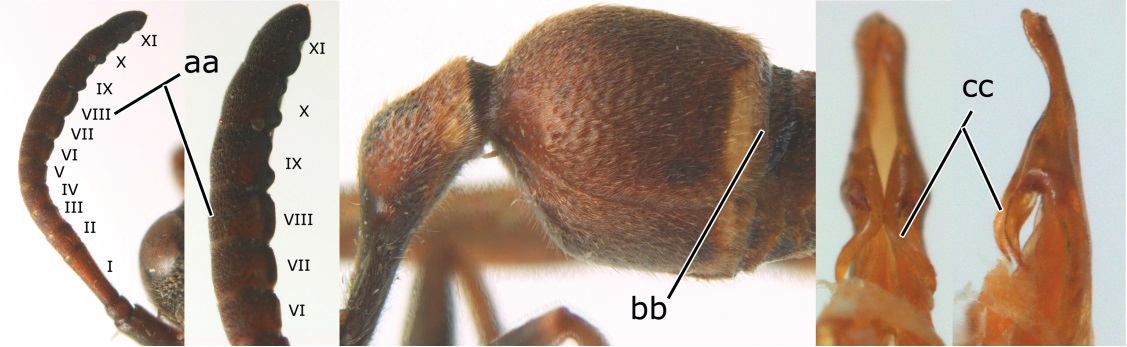	
9	Preapical part of second tergite distinctly swollen (a); lateral profile of second sternite nearly straight anteriorly (b); mesoscutum entirely black (c)	10
	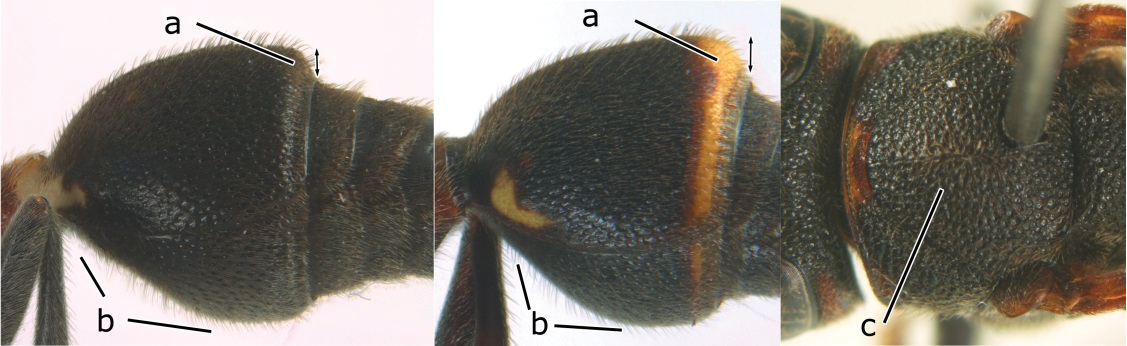	
–	Preapical part of second tergite flat (aa); lateral profile of second sternite evenly curved anteriorly (bb); colour of mesoscutum variable (cc)	11
	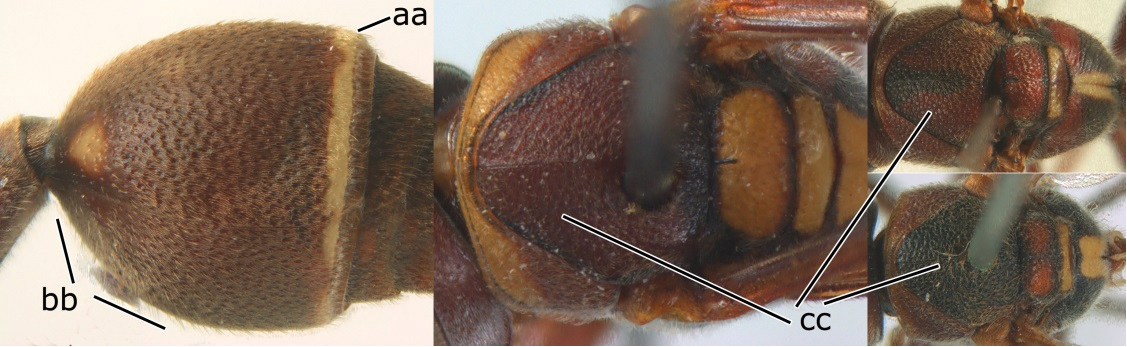	
10	Female clypeus yellow with a black arrow-shaped mark (a); second tergite with yellow apical band and strongly swollen medio-dorsally in lateral view (b); male genitalia: proximal margin of penis abruptly contracted in dorsal view and with a large hook in ventral view (c)	*Ropalidia artifex* (de Saussure, 1854)
	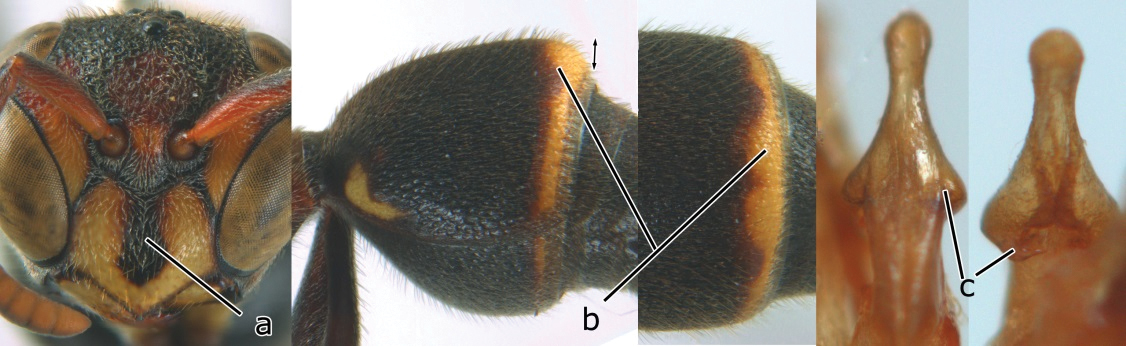	
–	Female clypeus largely black and apically yellow (aa); second tergite largely black and dorsally relatively weakly swollen in lateral view (bb); male genitalia: proximal margin of penis gradually contracted in dorsal view and with a comparatively small hook in ventral view (cc)	*Ropalidia parartifex* Tan & van Achterberg, sp. n.
	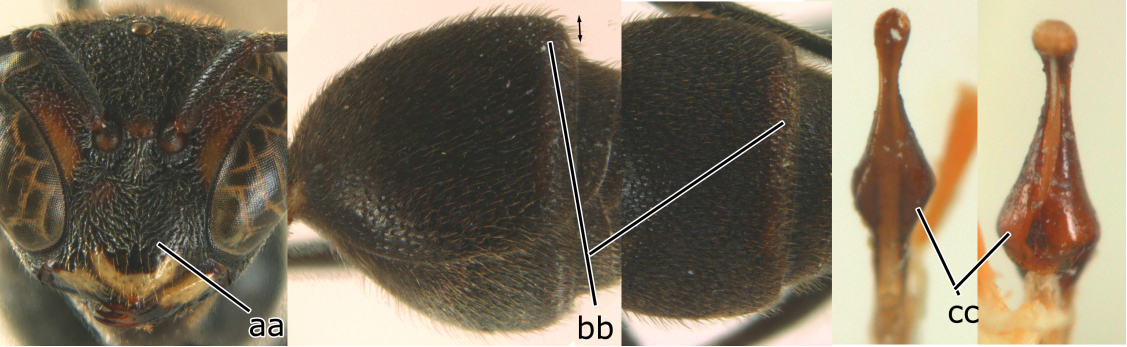	
11	Female clypeus reddish or blackish brown and yellow apically (a); gena reticulate-punctate (b); metapleuron black or with reddish brown patch (c); male genitalia: proximo-dorsal margin of penis gradually contracted (d)	12
	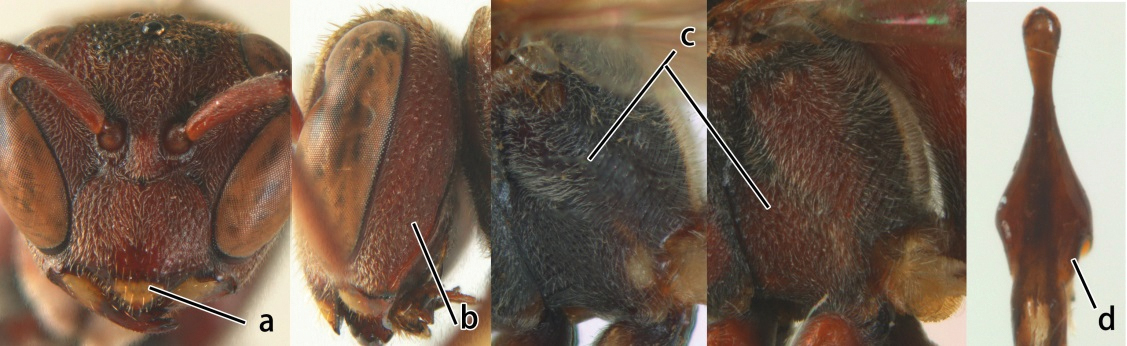	
–	Female clypeus yellow laterally (aa); gena finely punctate (bb); metapleuron generally with large yellow patch (cc); male genitalia: proximo-dorsal margin of penis abruptly contracted (dd)	13
	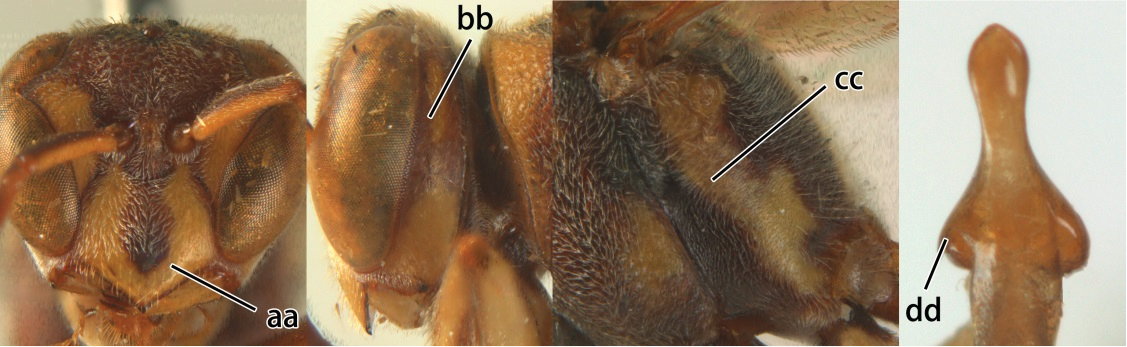	
12	Mesoscutum with two separate reddish brown patches (a); tergite and sternite of second metasomal segment fused and suture indistinct except posteriorly (b); male genitalia: proximo-ventral margin of penis valve hardly projecting (c)	*Ropalidia hongkongensis* (de Saussure, 1854)
	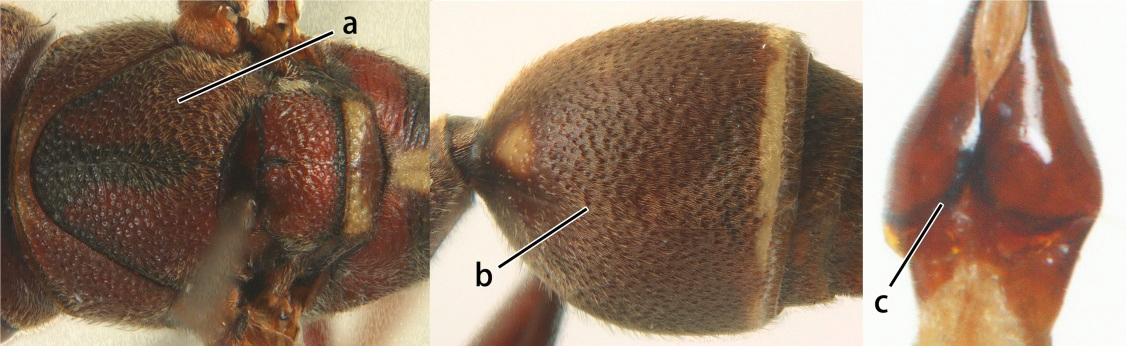	
–	Mesoscutum entirely black (aa); tergite and sternite of second metasomal segment separated and suture complete (bb); male genitalia: proximo-ventral margin of penis valve with a distinct projection (cc)	*Ropalidia rufocollaris* (Cameron, 1900)
	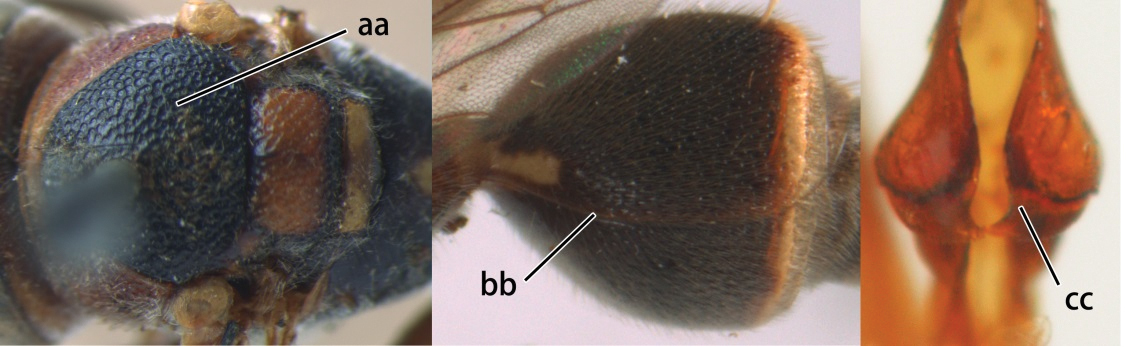	
13	Metapleuron ventrally punctate (a); female clypeus reddish brown with two lateral yellow spots (b); first metasomal segment reddish brown baso-laterally, without yellow pattern basally (c); second sternite usually without yellow spots (d); male antenna comparatively robust and serrate (e); male genitalia: proximo-ventral margin of penis valve with a relatively sharp hook-like projection (f)	*Ropalidia mathematica* (Smith, 1860)
	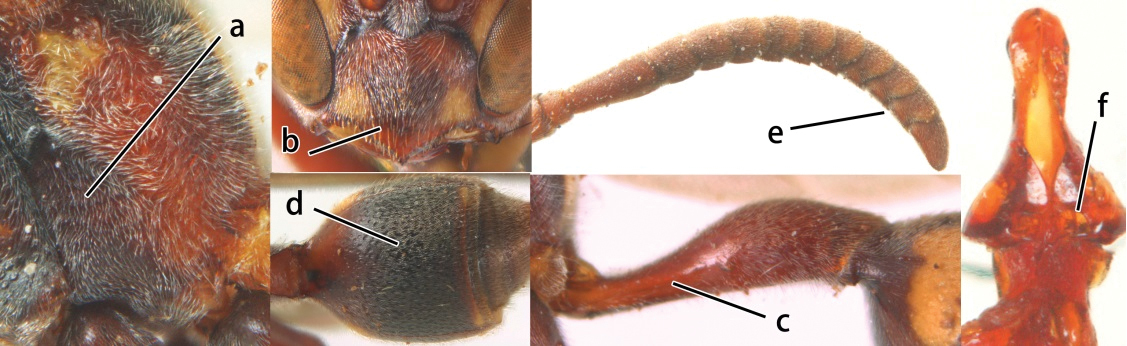	
–	Metapleuron ventrally smooth (aa); female clypeus yellow with a dark arrow-shaped mark medially (bb); first segment partly yellow baso-laterally (cc); second sternite usually with a pair of large yellow spots (dd); male antenna comparatively slender and hardly serrate (ee); proximo-ventral margin of penis valve evenly curved, without a hook-like projection (ff)	*Ropalidia stigma* (Smith, 1858)
	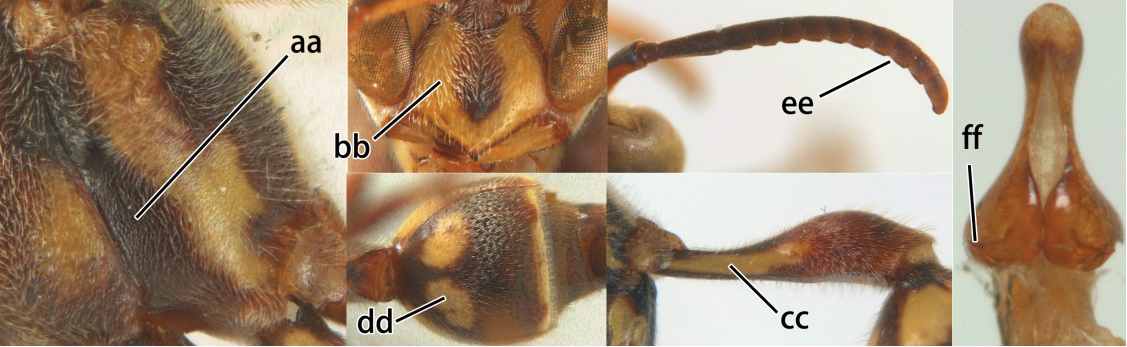	
14	Fore wing with two submarginal cells (a); OOL at most twice as long as POL or shorter (b)	*Ropalidia bicolorata* van der Vecht, 1962
	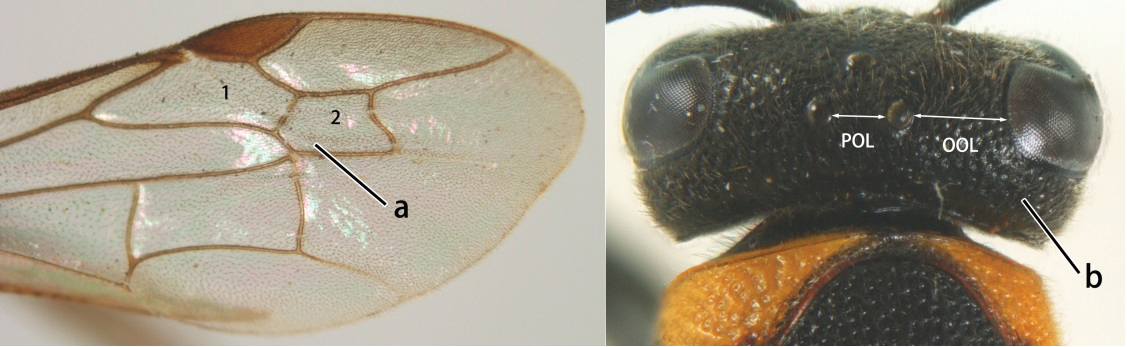	
–	Fore wing with three submarginal cells (aa); OOL at least 3 × as long as POL or longer (bb)	15
	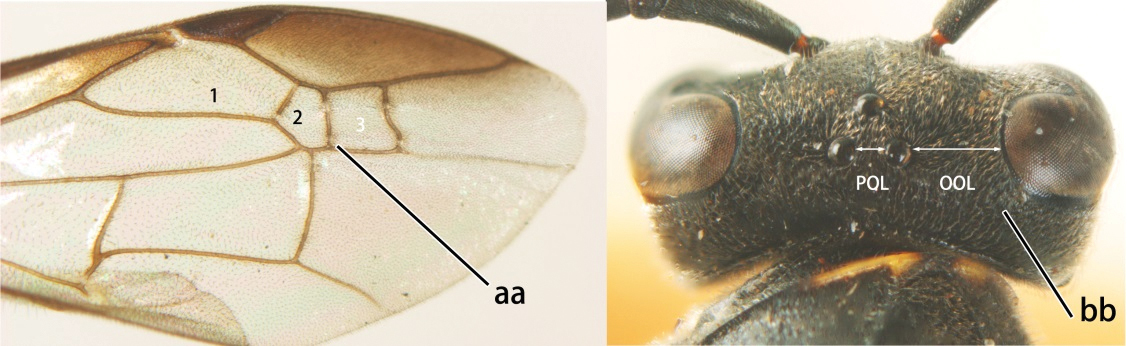	
15	Length of body 10–11 mm (measured from head to end of second metasomal tergite); pterostigma blackish brown and marginal cell entirely dark brown (a); mesoscutum and second metasomal tergite entirely black (b); mesopleuron (except antero-ventrally) coarsely punctate (c)	16
	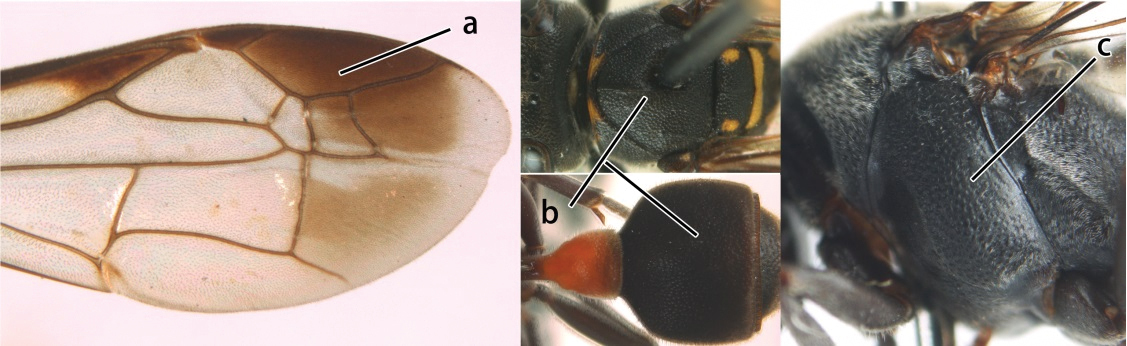	
–	Length of body 7–8 mm (measured from head to end of second metasomal tergite); pterostigma brown and ventral half of marginal cell subhyaline (aa, in *Ropalidia cyathiformis* anterior half of marginal cell subhyaline); mesoscutum generally with two yellow stripes and second tergite generally with pair of yellow spots and apical band (bb); mesopleuron weakly punctate (cc)	18
	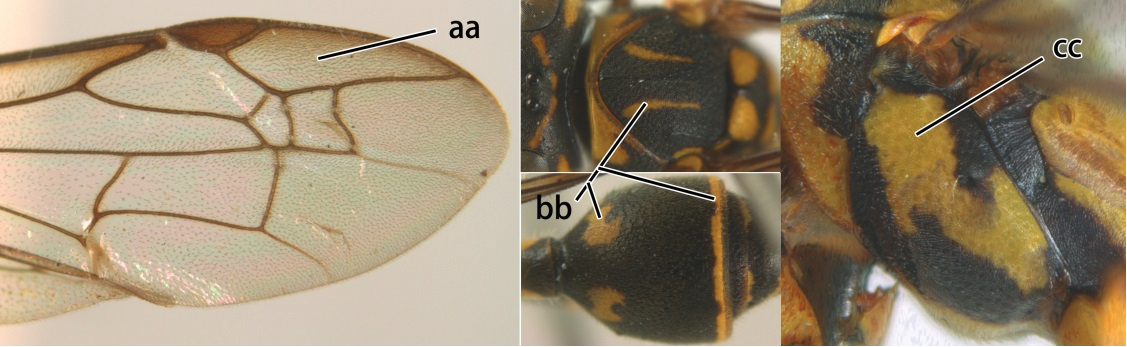	
16	Female clypeus coarsely and densely punctate (a); propodeum dull and distinctly punctate-striate (b); first metasomal segment black with pair of apical spots (c); second metasomal segment somewhat oblique with sternite shorter than tergite (d)	17
	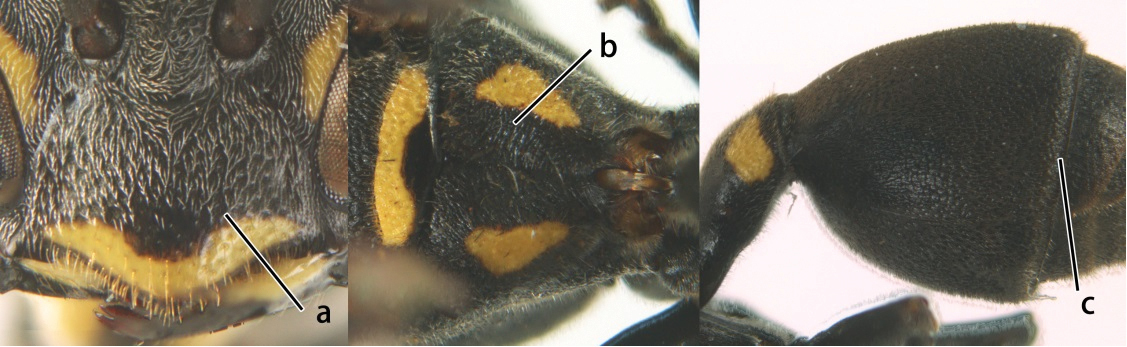	
–	Female clypeus finely punctate (aa); propodeum shiny and smooth, at most very finely striate (bb); first metasomal segment entirely orange (cc); second segment oblique with sternite distinctly larger than tergite (dd)	*Ropalidia sumatrae* (Weber, 1801)
	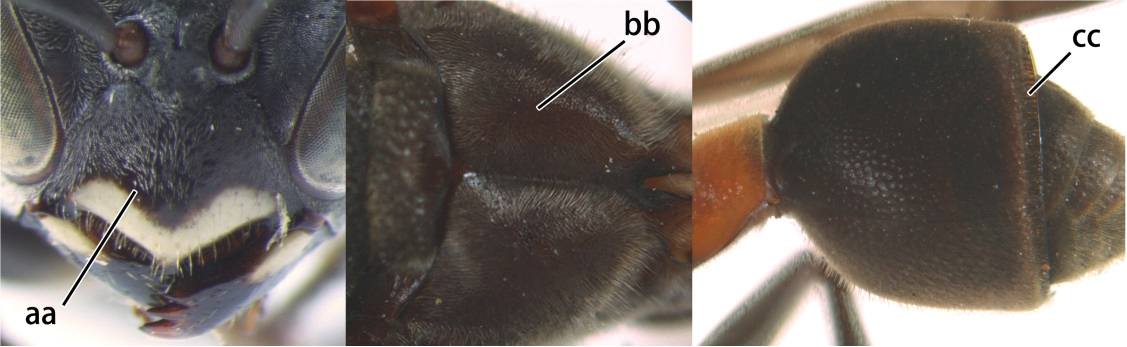	
17	Ventral half of occipital carina strongly widened, its maximum width nearly one-third genal width (a); pronotum with two small yellow spots medio-dorsally (b); scutellum and metanotum black with yellow pattern (c); propodeum with pair of large yellow patches (d); subapical spots of first tergite yellow and large (e)	*Ropalidia obscura* Gusenleitner, 1996, rec. n.
	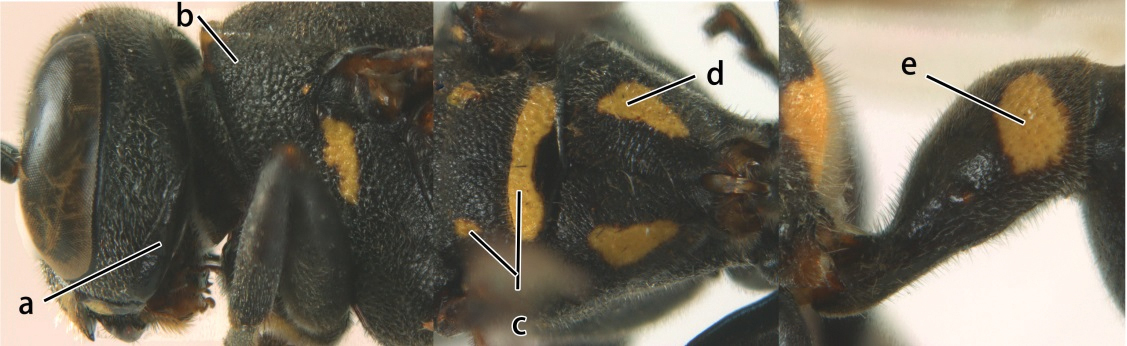	
–	Occipital carina hardly or not widened (aa); pronotum reddish brown medio-dorsally (bb); scutellum and metanotum reddish brown (cc); propodeum black (dd); apical spots of first tergite reddish brown and large (ee)	*Ropalidia scitula* (Bingham, 1897), rec. n.
	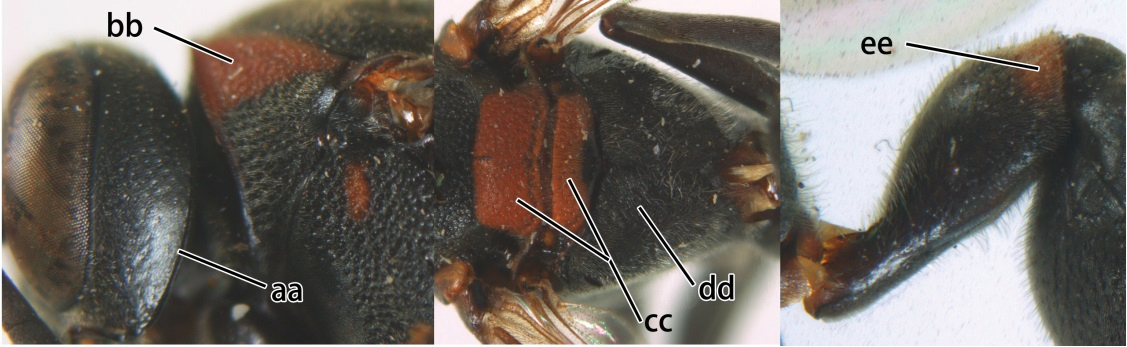	
18	Head reddish brown and with yellow patches near its occipital carina, and temple distinctly shorter than eye in dorsal view (a); pterostigma pale brown and basal half of marginal cell subhyaline (b); first metasomal tergite strongly widened in lateral view, its length less than 1.5× its height (c); male: flagellum more or less serrate ventrally and terminal flagellomere weakly curved (d)	*Ropalidia cyathiformis* (Fabricius, 1804), rec. n.
	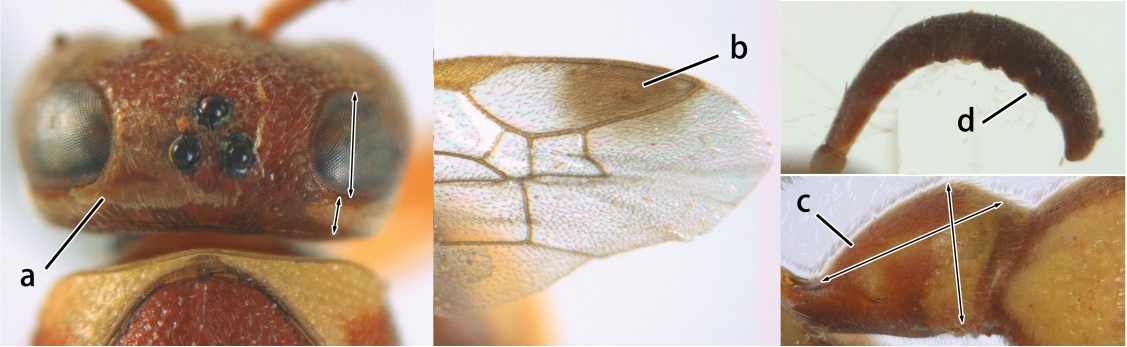	
–	Head black with yellow spots and temple as long as eye in dorsal view (aa); pterostigma dark brown and posterior half of marginal cell subhyaline (bb); first metasomal tergite weakly widened in lateral view, its length more than 2.2× its height (cc); male: flagellum slightly serrate and terminal flagellomere bullet-shaped (dd)	19
	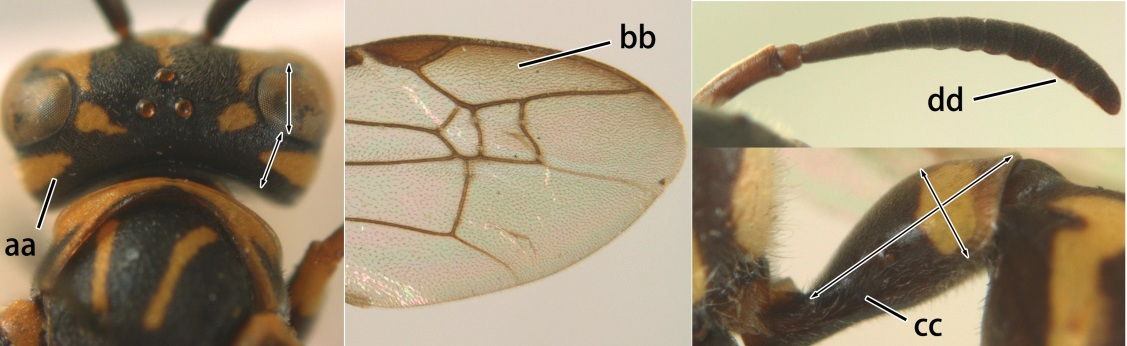	
19	Vertex, frons, mesoscutum and scutellum shiny, impunctate (a); mesopleuron largely yellow and without distinct coarse punctures (b); second metasomal sternum yellow on basal two-thirds (c)	*Ropalidia opifex* van der Vecht, 1962
	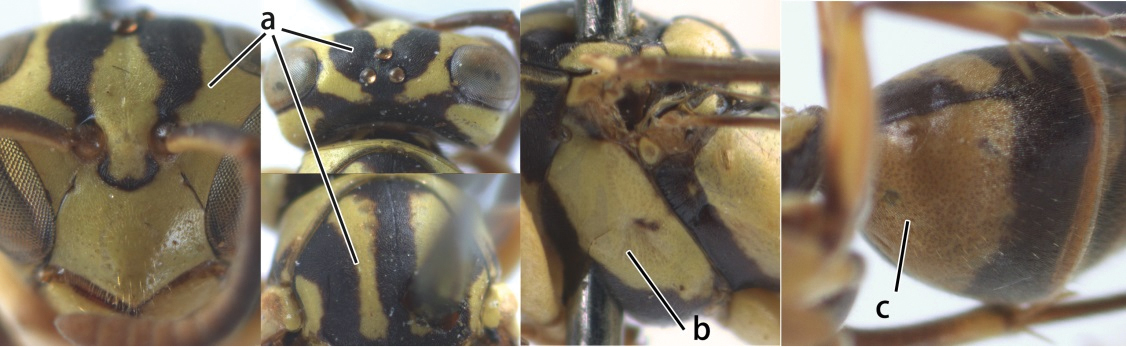	
–	Vertex, frons, mesoscutum and scutellum dull, finely punctulate with rather large, shallow, flat-bottomed punctures (aa); mesopleuron black with less extensive yellow pattern (bb, bb’) and with distinct coarse punctures (bb, bb’); second metasomal sternite black with small yellow spots (cc), but more extensively yellow in *Ropalidia ornaticeps* (cc’)	20
	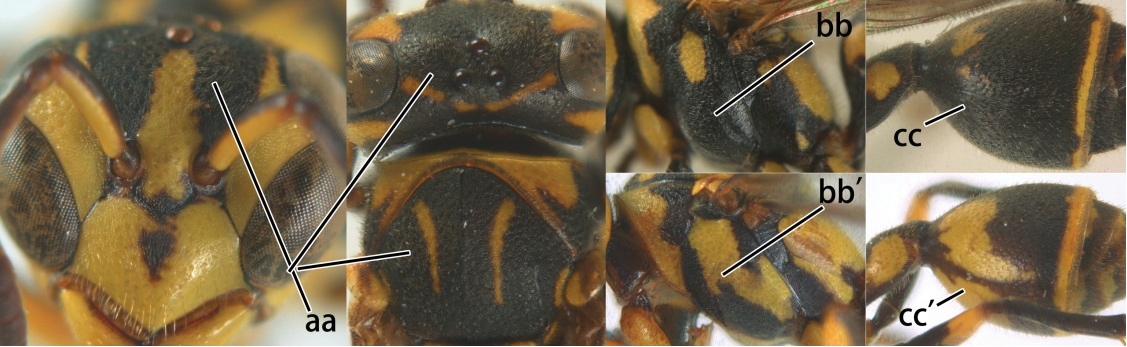	
20	Occipital carina sinuate near middle level of eye (a); clypeus yellow with an isolated black medial spot (b); apical part of first metasomal tergite comparatively wide (beyond spiracle slightly wider than long) and spiracle invisible in dorsal view (c); basal slender part of first metasomal tergite rather short in lateral and ventral view, rising directly from posterior end of reception of propodeal suspensory ligament (d)	*Ropalidia flavopicta* Smith, 1857
	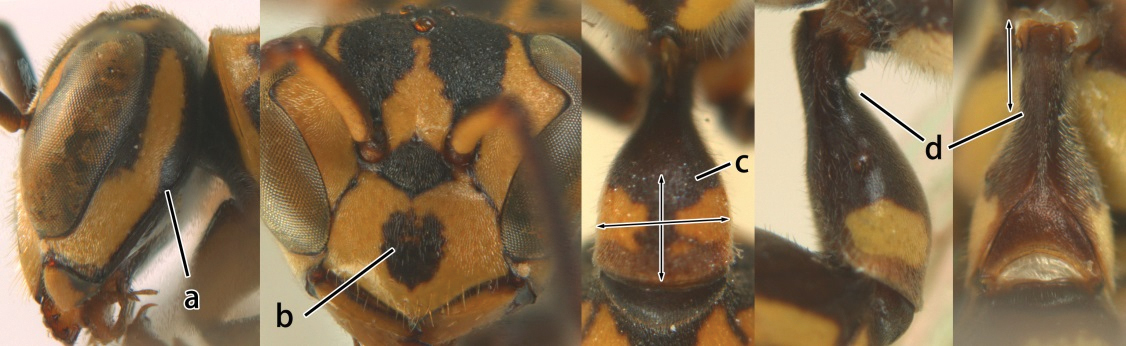	
-	Occipital carina evenly curved near middle level of eye (aa); clypeus entirely yellow (bb) or a large black spot (bb’); apical part of first metasomal tergite comparatively slender (beyond spiracle longer than wide or quadrate) and spiracle visible in dorsal view (cc); basal slender part of first metasomal tergite longer in lateral and ventral view, rising further away from propodeal suspensory ligament (dd)	21
	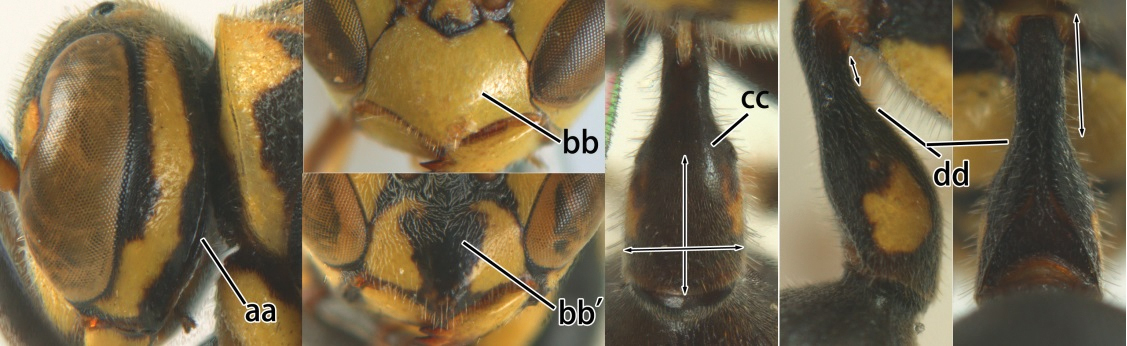	
21	Gena yellow, except upper half of occipital carina black (a); clypeus entirely yellow (b); mesopleuron with extensive yellow pattern (c) as second sternite (d)	*Ropalidia ornaticeps* (Cameron, 1900), rec. n.
	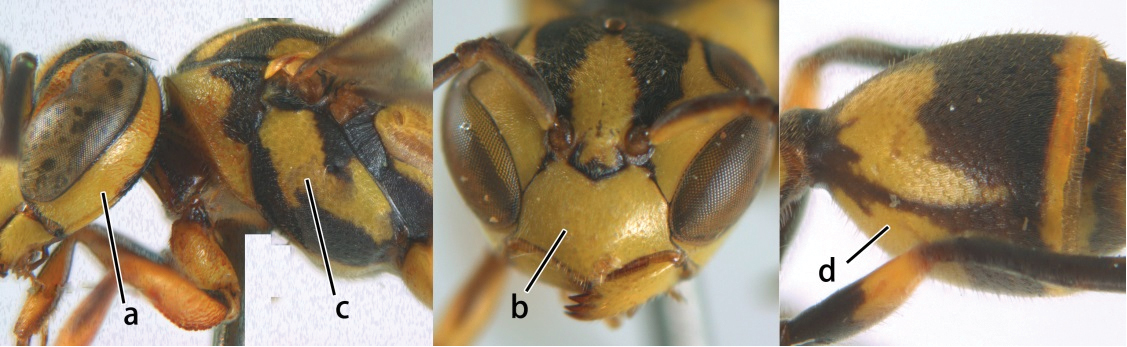	
-	Gena mainly black anteriorly and posteriorly, but medially yellow (aa); clypeus with a large triangular black medial spot (bb); mesopleuron with restricted yellow pattern (cc); second sternite black except a narrow yellow band apically (dd)	*Ropalidia malaisei* van der Vecht, 1962, rec. n.
	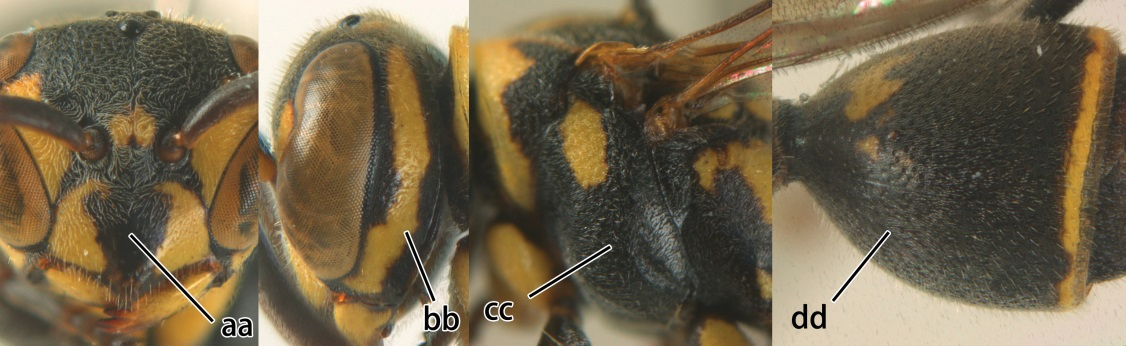	

## Distribution records and taxonomic remarks

### Ropalidia artifex

(de Saussure, 1854) rec. n.

http://species-id.net/wiki/Ropalidia_artifex

Figure 1A

Icaria artifex de Saussure, 1854: 236. Type locality: Java.
Ropalidia artifex ; Dover 1931: 257.
Ropalidia artifex artifex ; van der Vecht 1941: 110.
Ropalidia artifex fuscata van der Vecht 1941: 136. Type locality: N. Sumatra.

#### Specimens examined.

CHINA: 3 ♀ (NWUM), Yaoqu, Mengla, Yunnan, 28–30.ix.2010, JL Tan; 1 ♀ (NWUM), Longmen, Shangyong, Yunnan, 26.ix.2010, JL Tan; 1 ♀ (ZJUH), Jinghong Forestry Park, Yunnan, 31.vii.2003, J Lu (no. 20045916); 5 ♀ (ZJUH), Fugong, Yunnan, 24.viii.2003, Q Li (no. 20046662, -4, -6, -8, -70); 1 ♀ (ZJUH), Mt. Youle, Yunnan,17.iv.1981, JH He (no. 811876); 1 ♀ (ZJUH), Shuangjiang, Yunnan, 21.iv.1981, coll. JH He (no. 813609); 2 ♀ + 8 ♂ (ZJUH), Tongzhong, Shiwandashan, Guangxi, 3.xii.2001, ZF Xu (no. 20029971–4, -77–79, -80–82). INDONESIA: 2 ♀ (RMNH), W. Java: Djampang Tengah, 1634, M Walsh; 1 ♂ (RMNH), Udjung Kulon, Tjidaon, Java, 29.xi.1951, AMR Wegner. VIETNAM: 1 ♀ (RMNH), S. Vietnam: Dak Lak, Chu Yang Sin N.P., 50 km S. Buon Ma Thuot, Krong K’Mar, 550–600 m, 23–25.x.2005, near rainforest, C van Achterberg & R de Vries; 1 ♀ (RMNH), id., but 590–840 m, 22–26.x.2005, Malaise traps 13–23; 1 ♂ (RMNH), N. Vietnam: Viet Try, Thuong Cuu, near Thanh Son, 20°59'N, 105°8'E, 350–400 m, 12–16.x.1999, R de Vries. MYANMAR: 8 ♀ + 1 ♂ (RMNH), S. Shan state, Burma, 1500 m, Taunggyi, 1.viii–13.x.1934, R Malaise.

**Figure 1.**
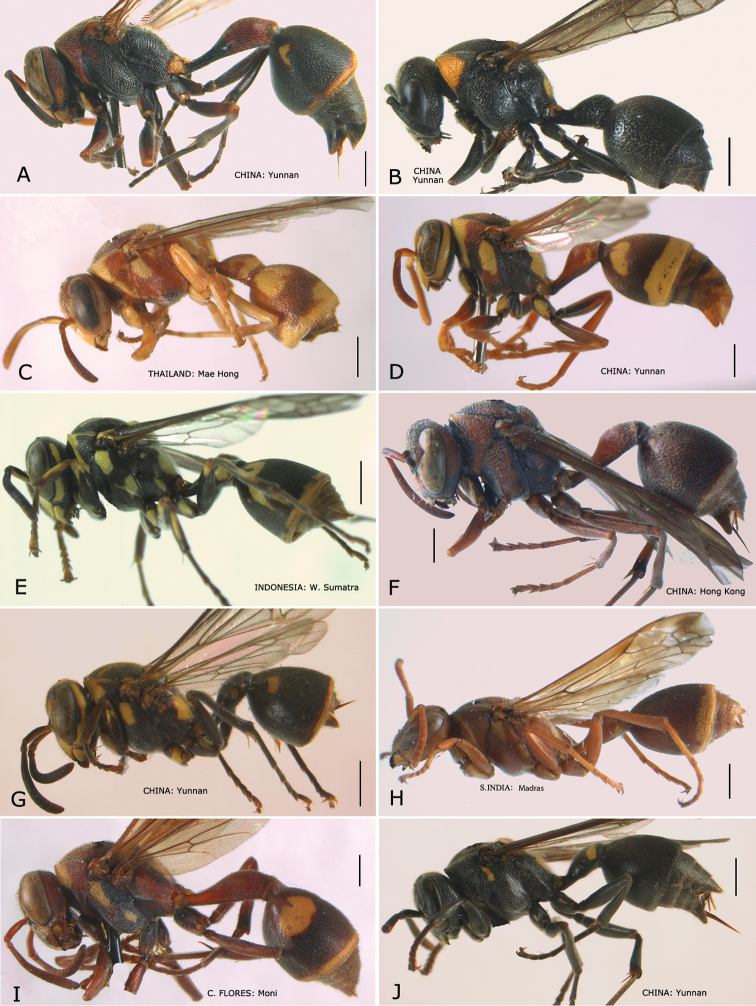
*Ropalidia* spp., habitus (♀). **A** *Ropalidia artifex* (de Saussure, 1854) **B** *Ropalidia bicolorata* van der Vecht, 1962 **C** *Ropalidia cyathiformis* (Fabricius, 1804) **D** *Ropalidia fasciata* (Fabricius, 1804) **E** *Ropalidia flavopicta* (Smith, 1857) **F** *Ropalidia hongkongensis* (de Saussure, 1854)**G** *Ropalidia malaisei* van der Vecht, 1962 **H** *Ropalidia marginata* (Lepeletier, 1793) **I** *Ropalidia mathematica* (Smith, 1860) **J** *Ropalidia obscura* Gusenleitner, 1996.

#### Remarks.

Some specimens have the apical margin of the second metasomal tergite flattened, not convex as in typical specimens. In the species *Ropalidia artifex*, there are two subspecies e.g. *Ropalidia artifex artifex* and *Ropalidia artifex fuscata* van der Vecht 1941. Up to date, no distinct morphological differences between them were found, and it is hard to formally treat them as two distinct species (van der Vecht 1962, Nguyen et al. 2006). The Chinese specimens belong all to *Ropalidia artifex artifex*.

#### Distribution.

China (Yunnan, Guangxi); Borneo; Java; Myanmar; Malay Peninsula; Sumatra; Vietnam (Kojima and Carpenter 1997 and updated to 2006, Nguyen et al. 2006).

### Ropalidia bicolorata

van der Vecht, 1962

http://species-id.net/wiki/Ropalidia_bicolorata

Figure 1B

Paraicaria bicolor Gribodo, 1892: 249 (preoccupied by *Ropalidia bicolor* (Smith 1865)). Type locality: Myanmar (Chan Yoma).
Ropalidia bicolorata bicolorata van der Vecht, 1962: 38.
Ropalidia bicolorata parvula van der Vecht, 1962: 38, 39. Type locality: N. Borneo (Bettotan near Sandakan). Syn. by Nguyen et al. 2006.
Ropalidia bicolorata shiva Das & Gupta, 1984: 428 (nomen nudum); 1989: 153. Type locality: India (Tripura). Syn. by Kojima et al. 2007.

#### Specimens examined.

1 ♀ (RMNH), syntype of *Paraicaria bicolor*, Chan Yoma, with a red handwritten label: *Paraicaria bicolor* det. Gribodo. MYANMAR: 1 ♀ (RMNH), Burma, S. Shan States, 1500 m, Taunggyi, i.viii.-22.ix.1934, R Malaise. CHINA: 9 ♀ (NWUM), Longmen, Shangyong, Yunnan, 26–27.ix.2010, JL Tan; 2 ♀ (NWUM), Yaoqu, Mengla, Yunnan, 28–30.ix.2010, JL Tan; 1 ♀ (GSFA), Xishuangbanna, Wangtianshu, Yunnan,23.iv.2002, SP Sun; 3 ♀ (ZJUH), Ruili, Yunnan, 29.iv.1981, JH He, no. 811457, 811458, 811458; 1 ♀ (ZJUH), Mengxiu, Ruili, Yunnan, 2–6.v.1981, JH He, no. 813071; 1 ♀ (ZJUH), Menglian, Yunnan, 19.iv.1981, JH He, no. 812639. THAILAND: 1 ♀ (RMNH), Thailand, Doi Inthanon, 8.i.1958, Umesao. MALAYSIA: 1 ♀ (RMNH), N. Borneo: Bettotan, near Sandakan, 26.vii.1927 (paratype of *Ropalidia bicolorata parvula* van der Vecht, 1962).

#### Remarks.

Among the specimens from China, there are two colour forms, *parvula* and the nominate species *bicolorata*.

#### Distribution.

China (Yunnan); Borneo; India; Malaysia; Myanmar; Thailand; Vietnam. (Kojima and Carpenter 1997 and updated to 2006, Nguyen et al. 2006, Kojima et al. 2007).

### Ropalidia binghami

van der Vecht, 1941

http://species-id.net/wiki/Ropalidia_binghami

Icaria sumatrae ; Bingham, 1897: 387 (misidentification).
Ropalidia binghami van der Vecht, 1941: 113. Type locality: Thailand (Siam).
Ropalidia binghami binghami ; van der Vecht 1962: 8.
Ropalidia sumatrae sumatrae ; Lee 1982: 88, 1985: 50.

#### Specimens examined.

1 ♀, Luang Prabang, Hat Thoun, Siam, 10. xi.1917, RV De Salvaza (holotype, BMNH) 1♂, Mergui, Tenasserim, May 1890, CT Bingham (allotype, BMNH. “This specimen bears a label in Bingham’s handwriting: “*Icaria cotonata* White ♀”).

#### Remarks.

No specimens from China were available, but this species is included in the key because it has been listed for China by Lee (1982, 1985) as *Ropalidia (Anthreneida) sumatrae sumatrae* (Weber). Considering the description and illustration of Lee (1982, 1985), we agree with Kojima and Carpenter (1997) and Kojima (2006) that it most likely concerns *Ropalidia binghami* van der Vecht, 1941.

#### Distribution.

China (Guangdong); Myanmar; Thailand. (Kojima and Carpenter 1997 and updated to 2006, Lee 1982, 1985).

### Ropalidia birmanica

van der Vecht, 1962

http://species-id.net/wiki/Ropalidia_birmanica

Ropalidia taiwana birmanica van der Vecht, 1962: 23. Type locality: Myanmar.
Ropalidia birmanica : Kojima et al. 2007: 382.

#### Specimens examined.

MYANMAR (specimens collected by R. Malaise are paratypes of *Ropalidia taiwana birmanica*): 1 ♂ + 1 ♀ (RMNH), S. Shan States, road 40 km. E. of Tannggyi, 25.ix.–13.x.1934, R Malaise; 2 ♀ (RMNH), Burma, S. Shan States, 1500 m, Tannggyi, 1.viii–22.ix.1934, R Malaise; 2 ♀ (RMNH), N. East Burma, Sadon, 1200 m, 28.vi–5.vii.1934, R Malaise; 1 ♀ (RMNH), Sukii, 75 km, E. of Mouimein, Tenasserim, 600 m, 27–31.x.1934, R Malaise; 2 ♀ (RMNH), Nam Tamai valley, Upper Burma, 3000 ft, 27°42'N, 97°54'E, 26.viii.1938, R Kaulback, B.M. 1938–741. CHINA: 1 ♀ (ZJUH), West Mt. Tianmu, Lin’an, Zhejiang, vi.1994, no. 940007, JH He; 1 ♀ (ZJUH), Mt. Jiulong, Huishui, Guizhou, 11.vi.2010, JL Tan.

#### Distribution.

China (Guizhou, Fujian, Zhejiang), Myanmar. (Kojima and Carpenter 1997 and updated to 2006, Kojima et al. 2007).

### Ropalidia cyathiformis

(Fabricius, 1804) rec. n.

http://species-id.net/wiki/Ropalidia_cyathiformis

Figure 1C

Eumenes cyathiformis Fabricius, 1804: 289. Type locality: Java.
Ropalidia cyathiformis ; van der Vecht 1941: 104.

#### Specimens examined.

INDONESIA: 1 ♀ (RMNH), Java, Malang, 11.iv.1930, JG Betrem. CHINA: 1 ♀ (NWAM), Xishuangbanna, Mengla, Yunnan, 20.iv.1982, JR Zhou & SM Wang.

#### Distribution.

China (Yunnan); Bali; India; Java; Lombok; Nepal; Malay Peninsula; Myanmar; Philippine Islands; Sumba, Sulawesi, Sri Lanka; Thailand; Vietnam. (Kojima and Carpenter 1997 and updated to 2006, Kojima et al. 2007).

### Ropalidia fasciata

(Fabricius, 1804)

http://species-id.net/wiki/Ropalidia_fasciata

Figure 1D

Eumenes fasciata Fabricius, 1804: 290. Type locality: Java.
Polistes bioculata Fabricius, 1804: 278. Type locality: Nova Cambria.
Icaria picta de Saussure, 1854: 238. Type locality: India (Le Bengale).
Ropalidia fasciata ; van der Vecht 1959: 245.

#### Specimens examined.

1 ♂ (ZMUC), with three handwritten labels “Lectotype, J.v.d.Vecht, 1957”, “*Ropalidia fasciata* (F.) ♂ = *picta* (Sauss.) det. v d Vecht, 1957”, “*E: fasciata*, O. Java”, and a red label: “Type”, ZMUC 00241458. CHINA: 2 ♀ (ZJUH), Songtao Reservoir, Hainan, 17.vii.2002, ZF Xu (no. 20029573–4); 2 ♂ + 7 ♀ (CATAS), Nada, Danzhou, Hainan, N 19°05'17", E109°34'50", 143 m; 1 ♀ (NWUM), Yaoqu, Mengla, Yunnan, 30.ix.2010, JL Tan; 1 ♂ (NWUM), Longmen, Shangyong, Yunnan, 27.ix.2010, JL Tan; 1 ♀ + 2 ♂ (ZJUH), Kaiyuan, Yunnan, v.1978, no. 780497, 780497, 780496, Y Huang; 1 ♀ (ZJUH), Kaiyuan, Yunnan,1984, no. 841202, YC Liao; 1 ♀ (ZJUH), Longchuan, Yunnan, 1981, no. 814945, SL Tao; 1 ♀ (ZJUH), Yunjiang, Yunnan, 4.iv.1981, no. 811491, JH He; 1 ♀ (RMNH), Canton (= Guangzhou); 1 ♀ (RMNH), Canton, 12.xi.1932; 2 ♀ (ZJUH), Fengkai, Guangdong, 16–18.v.1992, no. 921925, JH He; 1 ♀ (RMNH), Foochow (= Fuzhou), Kellogg; 2 ♂ (RMNH), Canton, 10.xi.1932, O Piel, no. 5.13.10.61, (with a handwritten label: “*Ropalidia variegata*, det. O Piel, 1935”); 4 ♀ (RMNH), N. Sanya, Gainan, Formosa, ii.1909, (labelled: *Ropalidia picta* Sauss., det. J v d Vecht ’33); 8 ♀ (RMNH), Anping, Formosa, vii.1911, H Sauter (labelled: *Icaria bioculata*, det. Schulthess and *Ropalidia picta* (Sauss.), det. J v d Vecht, 1940); 2 ♀ (RMNH), Taihorin, Formosa, vii.1911, H Sauter, (labelled: *Ropalidia variegata*, det. Schulthess); 6 ♂ + 1 ♀ (RMNH), id., but 7.xii.1911; 4 ♂ (RMNH), id., but labelled “*Ropalidia picta* (Sauss.), det. J v d Vecht, 1940”; 1 ♂ (RMNH), Formosa, Taihorinsho, H Sauter; 1 ♂ (ZJUH), Longquan, Zhejiang, 20.vii.1965, no. 65053.6, JH He & DD Jin; 1 ♀, (ZJUH) Jingning, Zhejiang, vii.1994, no. 943782, SF Ye; 1 ♀ (ZJUH), Suichang, Zhejiang, 4. vii.1980, no. 833877; 1 ♀ (ZJUH), Mt. Jiulong, Suichang, Zhejiang, 18.vii.1994, no. 944048, JH He. MALAYSIA: 1 ♀ (RMNH), N. Borneo, Sabah, 6 km S of Nabawan, near old airstrip 116°.27'E, 5°.2'N, 400 m, 24.vi.1987, J Huisman.

#### Distribution.

China (Fujian, Guangdong, Hainan, Hong Kong, Taiwan, Yunnan, Zhejiang); Bali; Bangka; Borneo; Flores; India; Java; Kariman Djava I.; Myanmar; Nepal; Nias; Malay Peninsula; Sumatra; Timor; Palawan; Ryukyu Islands; Philippines (Palawan); Sri Lanka; Thailand; Vietnam. (Kojima and Carpenter 1997 and updated to 2006, Kojima et al. 2007, Barthélémy 2008).

### Ropalidia flavopicta

(Smith, 1857)

http://species-id.net/wiki/Ropalidia_flavopicta

Figure 1E

Icaria flavopicta Smith, 1857: 99. Type locality: Borneo.
Ropalidia flavopicta ; Bequaert 1918: 246.
Ropalidia flavopicta flavopicta ; van der Vecht 1962: 42.

#### Specimens examined.

MALAYSIA: 1 ♂ (RMNH), S. Sabah, near Long Pa Sia, c 1010 m, 25.xi.–8.xii.1987, Mal. trap, C van Achterberg; 1 ♂ (RMNH), S. Sabah, Beaufort, 0°20'S, 115°43'E, 1.iv.1987, J van Tol & J Huisman; 1 ♀ (RMNH), Sabah, Brumas NBT, 23–27.iv.1973, KM Guichard. INDONESIA: 1 ♀ (RMNH), W. Sumatra, Padangpanjang, 1.v.1988, 0°30'S, 100°26'E, R Hensen (labelled *Ropalidia flavopicta* (Smith, 1857) by J. Kojima in 1996).

#### Remarks.

No specimens from China were available in the present study, but this species was reported from China by Liu (1936–37). Van der Vecht (1962) recognized eight subspecies of *Ropalidia flavopicta*. The identification characters of the species *Ropalidia flavopicta* were not well enough defined before Kojima (1982, 1996a) re-examined the eight subspecies and concluded that eight valid species are involved. Therefore, the presence in China needs reconfirmation.

#### Distribution.

China (Hong Kong); Borneo; Malay Peninsula; Sumatra; Vietnam. (Liu 1936–37, van der Vecht 1962, Kojima 1996a, Kojima and Carpenter 1997 and updated to 2006, Nguyen et al. 2006)

### Ropalidia hongkongensis

(de Saussure, 1854)

http://species-id.net/wiki/Ropalidia_hongkongensis

Figure 1F

Icaria hongkongensis de Saussure, 1854: 239. Type locality: China (Hong Kong).
Ropalidia hongkongensis hongkongensis Das & Gupta, 1983: 418; Das and Gupta 1989: 111.
Ropalidia hongkongensis juncta van der Vecht, 1941: 141. Type locality: W. Java. Syn. by Nguyen et al. (2006).

#### Specimens examined.

CHINA: 1 ♂ (BMNH), Hummocks, Hainan, 24.v.1936, G. Ros, (allotype of *Ropalidia hongkongensis*); 1 ♀ (BMNH), Hongkong Peak, c. 1300 ft, 14–17.ix.1937, Miss Hurford, B.M. 1938–426; 9 ♀ + 5 ♂ (NWUM, RMNH), Yaoqu, Mengla, Yunnan, 28, 30.ix.2010, JL Tan; 2 ♀ (ZJUH), Hongmao Village, Yuanmen, Baisha, Hainan, 29.iv.2010, J.L. Tan; 1 ♀ + 2 ♂ (GSFA), Quannan, Jiangxi, 27.xi. & 1.xii.2010, SC Li; 1 ♂ (ZJUH), Mt. Yunji, Xinfeng, Guangdong, 19.vi.2002, no. 20029072, ZF Xu; 1 ♀ (ZJUH), Chengjia, Yangshan, Guangdong, 25.vii.2002, no. 20029350, ZF Xu; 1 ♀ (ZJUH), Shaoguan, Guangdong, 12.ix.1992, no. 921802, JH He; 1 ♀ (ZJUH), Fengkai, Guangdong,v.1992, no. 924296, XX Chen.

#### Distribution.

China (Guangdong, Hainan, Hong Kong, Jiangxi, Yunnan); Bangka; India; Java; Myanmar; Vietnam. (Kojima and Carpenter 1997 update to 2006, Nguyen et al. 2006, Kojima et al. 2007)

### Ropalidia malaisei

van der Vecht, 1962 rec. n.

http://species-id.net/wiki/Ropalidia_malaisei

Figure 1G

Ropalidia malaisei van der Vecht, 1962: 42, 65; Das and Gupta 1983: 427; 1989: 113, 151; Gusenleitner 1996: 16; Kojima 1996a: 325, 328, 340. Type locality: Myanmar.

#### Specimens examined.

MYANMAR: 4 ♂ (RMNH), N. East Burma: Sadon, 1200 m, 28.vi–5.vii.1934, R. Malaise (paratypes of *Ropalidia malaisei*). CHINA: 1 ♀ (NWUM), Longmen, Shangyong, Yunnan, 26.ix.2010, JL Tan; 20 ♀ (ZJUH, RMNH), Xishuangbanna Forestry Park, Yunan, 31.vii.2003 (no. 20045871, -74, -76, -77–82 (-79 in RMNH), -84–91, -93, -95), Q Jiang.

#### Remarks.

The yellow stripes of the mesoscutum are lacking in some individuals.

#### Distribution.

China (Yunnan), Myanmar. (Kojima and Carpenter 1997 and updated to 2006).

### Ropalidia marginata

(Lepeletier, 1836)

http://species-id.net/wiki/Ropalidia_marginata

Figure 1H

Vespa ferruginea Fabricius, 1793: 280 (junior primary homonym of *Vespa ferruginea* Gmelin, 1790). Type locality: India.
Ropalidia ferruginea ; Bequaert 1918: 247.
Epipona marginata Lepeletier, 1836: 541. Type locality: “Inde”.
Ropalidia marginata marginata ; van der Vecht 1941: 109, 117.
Ropalidia marginata indica van der Vecht, 1941: 121 (replacement name for *Vespa ferruginea* Fabricius, 1793).
Ropalidia marginata sundaica van der Vecht, 1941: 122. Syn. by Nguyen et al. 2006.
Icaria jucunda Cameron, 1898: 46.
Ropalidia marginata jucunda (Cameron, 1898): van der Vecht 1941: 104. Syn. by Nguyen et al. 2006.

#### Specimens examined.

VIETNAM: 1 ♀ (RMNH), S. Vietnam: Dak Lak, Chu Yang Sin N.P. 50 km S. Buon Ma Thuot, Krong K’Mar, 550–600 m, 23–25.x.2005, near rainforest, C van Achterberg & R de Vries; 1 ♂ (RMNH), S. Vietnam: Dông Nai, Cát Tien N.P., Head Quarters, 3.x.2005, at light, C van Achterberg & R de Vries. INDIA: 1 ♀ (RMNH), S. India, Madras State, Coimbatore, 1400 ft., v.1960, P Susai Nathan; 1 ♀ (RMNH), Madras, India; 1 ♂ (RMNH), India, Ajanda, 13.vii.1978, J Timor. INDONESIA: 1 ♀ + 1 ♂ (RMNH), Java, Malang, Kawi, iv.1933, JG Betrem. SRI LANKA: 1 ♂ (RMNH), Col. Dist., Colombo, 50 ft., museum gardens, 15.i.1977, KV Krombein & P Fernando.

#### Remarks.

No specimens from China were available in the present study, but this species was reported from China by Lee (1982, 1985) as *Ropalidia (Anthreneida) ferruginea* (Fabricius). However, Lee’s (1982, 1985) description and illustration are far from suffcient to define the species. Barthélémy (2008) recorded it from Hong Kong.

#### Distribution.

China (Guangdong, Hong Kong); Australia (Thursday Islands, Queensland); Bali; Bangka; Borneo; India; Java; Kariman Djava I.; Lombok; Malay Peninsula; Mariana Islands; Myanmar; New Britain; New Guinea; Pakistan; Palau Islands; Philippine Islands; Sri Lanka; Sulawesi; Sumatra; Sumba; Sumbawa; Talud Islands; Tukang Besi Islands; Vietnam; Volcano Islands. (Lee 1982, 1985, Kojima and Carpenter 1997 and updated to 2006, Kojima et al. 2007, Barthélémy 2008).

### Ropalidia mathematica

(Smith, 1860)

http://species-id.net/wiki/Ropalidia_mathematica

Figure 1I

Polybia mathematica Smith, 1860: 90. Type locality: Sulawesi (Makassar).
Ropalidia mathematica mathematica ; van der Vecht, 1941: 110.
Ropalidia mathematica binotata van der Vecht, 1941: 131. Syn. by Kojima et al. (2005).
Icaria nigroplagiata Cameron, 1900: 498.
Ropalidia mathematica nigroplagiata ; van der Vecht, 1941: 104. Syn. by Kojima et al. (2005).
Ropalidia mathematica sumbaensis van der Vecht, 1962: 20. Syn. by Kojima et al. (2005).

#### Specimens examined.

INDONESIA: 1 ♀ (RMNH), W. Flores, Rana Mese, 1300 m, 21.xi.1949, Dr. Bühler & Dr. Sutter (labeled as *Ropalidia mathematica mathematica* Smith, van der Vecht in 1956); 1 ♀ (RMNH), Moni, C. Flores, Wolowaru, 11.xi.1949, Dr. Bühler & Dr. Sutter; 16 ♀ + 7 ♂ (RMNH), Timor, Wiencke; 5 ♂ (RMNH), Batavia [= Jakarta], v.1908, E Jacobson; 1 ♂ (RMNH), Java, Mt. Gede, Tapos, 800 m, viii.1933, J v d Vecht; 2 ♀ (RMNH), W. Java, Pelabuan Ratu, 5–6.viii.1972, 0–50 m, J v d Vecht. SRI LANKA: 1 ♂ (RMNH), Col. Dist., Gamaha Botanic Garden, 28.i.1979, KV Krombein, PB Karunaratne, T Wijesinhe, S Siriwardane & T Gunawardane (labeled *Ropalidia marginata marginata* (Lep.) by van der Vecht in 1979).

#### Remarks.

No specimens from China were available in the present study, but the species was reported from China (Hong Kong) by Barthélémy (2006, 2008). However, according to his pictures, at the base of the first metasomal tergum is a pair of distinct yellow lateral stripes as in typical *Ropalidia stigma*. Therefore, the presence in China needs reconfirmation, but its presence in Vietnam and Thailand indicates that this species may occur in southern China.

#### Distribution.

?China (Hong Kong), India, Thailand, Vietnam, Bangka, Sumatra, Java, Kariman Djava Isl., Bali, Lombok, Sumbawa, Flores, Sumba, Timor, Sulawesi. (Kojima and Carpenter 1997 and updated to 2006, Kojima et al. 2007).

### Ropalidia obscura

Gusenleitner, 1996 rec. n.

http://species-id.net/wiki/Ropalidia_obscura

Figure 1J

Ropalidia obscura Gusenleitner, 1996: 15; Kojima and van Achterberg 1997: 10. Type locality: Thailand.

#### Specimens examined.

CHINA: 3 ♀ (NWUM), Mengla, Yaoqu, Yunnan, 27–30.ix.2010, JL Tan; 1 ♀ (NWUM), Longmen, Shangyong, Yunnan, 26.ix.2010, JL Tan; 1 ♀ (NWUM), Banna, Menglun, Yunnan, 5.x.2010, JL Tan. THAILAND: 1 ♀ (RMNH), 50 km SW Loei (17°20'N, 101°20'E), Phu Rua N. P, 14.vii.1986, R Hensen; (labeled *Ropalidia obscura* Gusenleitner, 1996, by J Kojima in 1996).

#### Distribution.

China (Yunnan), Thailand. (Kojima and Carpenter 1997 and updated to 2006).

### Ropalidia opifex

van der Vecht, 1962

http://species-id.net/wiki/Ropalidia_opifex

Figure 2A

Ropalidia opifex van der Vecht, 1962: 42; Richards 1978: 128; Lee 1982: 86; 1985: 46, 51; Kojima 1996a: 325, 328. Type locality: Malaya (Penang).

#### Specimens examined.

MALAYSIA: 1 ♀ (RMNH), Penang Hill, Penang, 2500 ft, 27.i.1959, H.T. Pagden (paratype of *Ropalidia opifex*). INDONESIA: 1 ♀ (RMNH), E. Borneo, Begen River, Tabang, 26.ix.1956, AMR Wegner.

**Figure 2.**
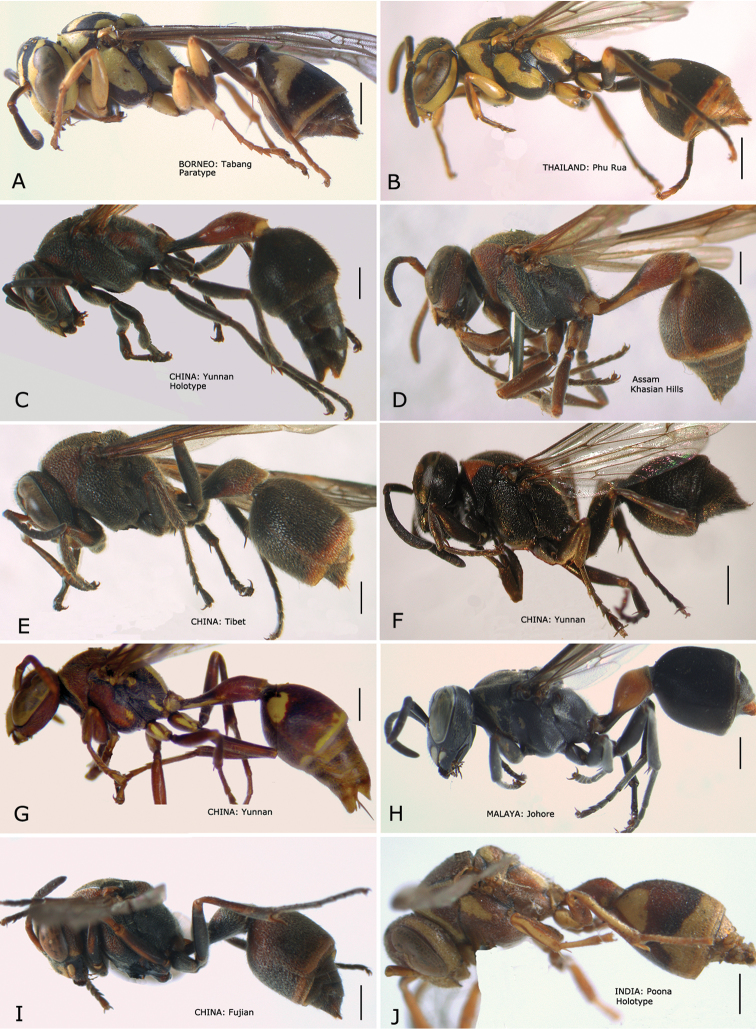
*Ropalidia* spp., habitus (♀). **A** *Ropalidia opifex* van der Vecht, 1962 **B** *Ropalidia ornaticeps* (Cameron, 1900) **C** *Ropalidia parartifex* Tan & van Achterberg, sp. n. (holotype) **D** *Ropalidia rufocollaris* (Cameron, 1900) **E** *Ropalidia santoshae* Das & Gupta, 1989 **F** *Ropalidia scitula* (Bingham, 1897) **G** *Ropalidia stigma* (Smith, 1858) **H** *Ropalidia sumatrae* (Weber, 1801) **I** *Ropalidia taiwana* Sonan, 1935 **J** *Ropalidia variegata* (Smith, 1852).

#### Remarks.

No specimens from China were available in the present study, but this species was reported from China by Lee (1982, 1985).

#### Distribution.

China (Yunnan); Borneo; Malay Peninsula. (Kojima and Carpenter 1997 and updated to 2006).

### Ropalidia ornaticeps

(Cameron, 1900) rec. n.

http://species-id.net/wiki/Ropalidia_ornaticeps

Figure 2B

Icaria ornaticeps Cameron, 1900: 496. Type locality: India.
Ropalidia flavopicta ornaticeps ; van der Vecht 1962: 49.
Ropalidia ornaticeps ; Yoshikawa et al. 1969: 167; Kojima 1996a: 325.

#### Specimens examined.

CHINA: 1 ♀ (CATAS), Nada, Danzhou, Hainan, 19°5'17"N, 109°34'50"E, 143 m. VIETNAM: 46 ♀ + 1 ♂ (RMNH, IEBR), Dông Nai, Cát Tien N.P., Mal. traps, x.2005 & iv.–v.2007, C van Achterberg & R de Vries; 1 ♂ (RMNH), Thua Thien Hué, Phong Dién N.R., 23.iii.–6.iv.2001, Mal. traps 6–9, C van Achterberg & R de Vries. THAILAND: 3 ♀, (RMNH), Chiang Khan, 17.vii.1986, R Hensen, with a label "Ropalidia ornaticeps (Cameron, 1900), det. J Kojima, 1996".

#### Remarks.

The clypeus is completely yellow, but sometimes with a small black spot; the occipital carina is variable, sometimes the carina is bent anteriorly as in *Ropalidia flavopicta*.

#### Distribution.

China (Hainan); Cambodia; India, Malay Peninsula; Myanmar; Thailand; Vietnam. (Kojima and Carpenter 1997 and updated to 2006, Kojima et al. 2007).

### Ropalidia parartifex

Tan & van Achterberg sp. n.

http://zoobank.org/F28CA4A3-0334-448E-B363-2AC1BB46DC0A

http://species-id.net/wiki/Ropalidia_parartifex

Figures 2C , 3

#### Holotype.

♀ (NWUM), CHINA: Longmen, Shangyong, Yunnan, 27.ix.2010, JL Tan. **Paratypes:** 2 ♀ + 1 ♂ (NWUM, RMNH), same data as holotype; 1 ♀ (GSFA), Xishuangbanna, Wangtianshu, Yunnan, 610 m, 23.iv. 2002, SP Sun; 2 ♂ (ZJUH), Jinghong Forestry Park, Yunnan, 31.vii.2003, no. 20045919, 20045938, J Lu; 2 ♀ (ZJUH), Mt. Youle, Yunnan,17.iv.1984, no. 811874, 811873, JH He; 1 ♀ + 2 ♂ (NWUM), Banna, Menglun Yunnan, 6.x.2010, JL Tan; 4 ♂ (NWUM), Yaoqu, Mengla, Yunnan, 28.ix.2010, JL Tan. THAILAND: 1 ♀ (RMNH), Chieng Dao, 19.1.1958, K. Yoshikawa (identified as *Ropalidia artifex* (Sauss.) var. by van der Vecht in 1960); 1 ♀ + 1 ♂ (RMNH), Siam, Chiangmai, 23.x.1922, F.4560, F.4571 (the female has the first tergite entirely black).

#### Diagnosis.

The new species is similar to *Ropalidia artifex*, but differs as follows: clypeus of female largely black and apically yellow (yellow with a black arrow-shaped mark in *Ropalidia artifex*); second metasomal tergum entirely black and strongly swollen subapically (with yellow apical band apically and weakly swollen subapically); male genitalia: proximal margin of penis gradually contracted in dorsal view and with a comparatively small hook in ventral view (abruptly contracted in dorsal view and with a large hook in ventral view).

#### Description.

♀, length of body (head + mesosoma + metasomal segments I–II) 11–12 mm, fore wing 10.5–12 mm.

**Figure 3.**
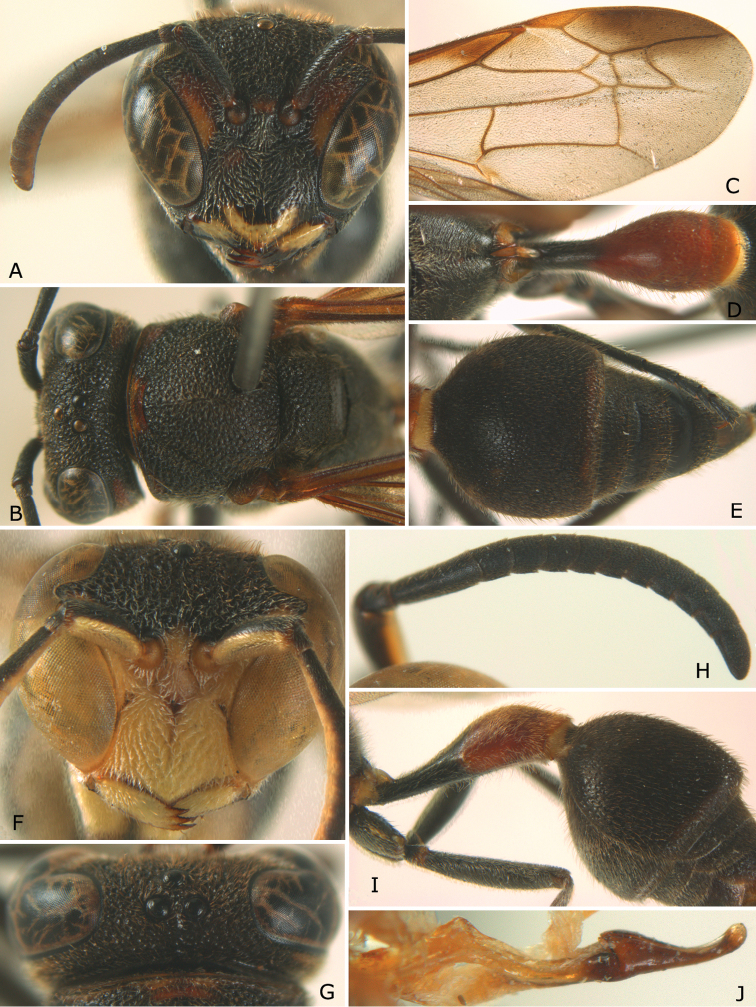
*Ropalidia parartifex* Tan & van Achterberg, sp. n. **A–E** Female (♀): **A** Head, frontal view **B** Head and mesosoma, dorsal view **C** Right fore wing **D** Propodeum and first metasomal segment, dorsal view **E** Second metasomal segment to the end, dorsal view **F–J** Male (♂): **F** Head, frontal view **G** Head, dorsal view **H** Right antenna **I** Metasomal, latral view **J** Penis, lateral view.

*Head*. Head in frontal view about 1.2 × as wide as high; in dorsal view 2.2 × as wide as long, straightly contract behind eye, emarginate posteriorly, about 1.1 × as broad as mesonotum (including tegulae). Gena in lateral view about 0.7 × as wide as eye; occipital carina present completely, slightly sinuate laterally (Fig. 2C). Posterior ocelli slightly closer to each other than to anterior ocellus; OOL (posterior ocellus-ocular distance):diameter of ocellus:POL (distance between the two posterior ocelli) = 10:4:3. Inner eye margins converging ventrally, about 1.25 × further apart at vertex than at clypeus. Clypeus weakly convex, pointed apically, nearly 1.1 × wider than high. Inter-antennal distance slightly shorter than antenna-ocular distance. Antenna about 4.0 mm long, gradually thickened apically; scape slightly curved, about 4.4 × as long as its apical width; third article slightly more than 3.3 × as long as its apical width, about as long as fourth, fifth and sixth articles combined; terminal article bullet-shaped, about 1.2 × as long as its basal width (Fig. 3A).

*Mesosoma*. Mesosoma robust, in dorsal view about 1.4× as long as wide (Fig. 3B). Pronotum in dorsal view broadly and weakly rounded anteriorly, with lateral sides diverging posteriorly in straight lines; ventral corner gradually narrowed (Figs 2C, 3B); pronotal carina raised entirely and sinuate laterally. Scutellum trapezoid, strongly convex. Posterior face of propodeum weakly convex, without median complete furrow, slightly depressed in front of orifice (Fig. 3D).

*Metasoma*. First segment elongate, about 3.5 × as long as high, 2.5 × as long as wide, weakly widening posteriorly from level slightly anterior to spiracle, widest at two thirds of its length and slightly contracted apically. Profile of second sternite in lateral view curved in anterior two thirds, with second tergite strongly swollen subapically (Fig. 3D, E).

*Sculpture*. Body covered with fine tomentum except in apical half of clypeus and with sparse setae. Clypeus, front, antero-ventral area of mesopleuron, metapleuron and sides of propodeum with scattered fine punctures; vertex, pronotum, mesoscutum, scutellum and postero-dorsal area of mesopleuron reticulate-rugose; posterior face of propodeum densely striate; first metasomal tergite shiny, with fine and sparse punctures on anterior half, and dense punctures on its posterior half; second–sixth metasomal segments dull, with dense punctures.

*Colour* (Fig. 2C). Body black to blackish brown with irregular reddish brown patches on the following parts: upper half of clypeus, ventral part of ocular sinus, gena, pronotum, mesopleuron antero-dorsally and propodeum laterally, but clypeus apically, mandible basally, tegula, propodeum valve and basal narrow part of second metasomal segment yellowish brown; apical half of first metasomal segment reddish brown. Coxa basally with irregular small yellow stripes. Fore wing slightly brown tinged with pterostigma yellowish brown; marginal cell and median cell apically dark brown (Fig. 3C).

*Male* (Figs 3F–J). Similar to female but head more transverse, in frontal view about 1.25 × as wide as high; in dorsal view 3.3× as wide as long, directly contracted behind eyes (Figs 3F, G). Antenna with 13 articles, third to terminal articles with slightly raised tyloids (Fig. 3H). Frons, clypeus, mandible, ventral antenna and antero-ventral area of mesopleuron yellow. Male genitalia (Fig. 3J): proximal margin of penis gradually contracted in dorsal view and curved slightly into a hook in ventral view.

#### Etymology.

The species name is derived from “para” (Latin for “near”) and “artifex”, because it is similar to *Ropalidia artifex*.

#### Distribution.

China (Yunnan); Thailand.

### Ropalidia rufocollaris

(Cameron, 1900)

http://species-id.net/wiki/Ropalidia_rufocollaris

Figure 2D

Icaria rufocollaris Cameron, 1900: 497. Type locality: India (Khasia Hills).
Ropalidia rufocollaris rufocollaris ; Das and Gupta 1983: 421; 1989: 125.
Ropalidia rufocollaris atrata van der Vecht, 1941: 139. Type locality: Thailand (Siam Doi Setep). Syn. by Nguyen et al. (2006).

#### Specimens examined.

CHINA: 1 ♀ (RMNH), Tibet, Khamba Jong, F.A.M. INDIA: 1 ♀ + 1 ♂ (RMNH), Khasia Hills, Assam; 1 ♀ (RMNH), British Sikkim, 200 m, valley at Tista Bridge, 8–15.xii.1934, R Malaise; 1 ♀ (RMNH), E. Himalayas, Pashok, 2000 ft, Darjiling district, 11.vi.16, LC Hartless; 1 ♀ (RMNH), India, Lushai Hills, Miso district, Thingsat, 2000–3500 ft, 9–10.ix.1960, F Schmid. MYANMAR: 1 ♂ (RMNH), Burma: Washaung, 20 km, East of Myitkyina, c 200 m, 14.vii.1934, R Malaise; 1 ♀ (RMNH), Nord East Burma: Punkatang, road Sadon-Myitkyina, 8.vii.1934, R Malaise.

#### Remarks.

Occipital carina is curved forward ventrally and the gena narrow ventrally; the propodeum has the yellow stripes merged, but are sometimes lacking.

#### Distribution.

China (Tibet); India; Laos; Myanmar; Thailand; Vietnam. (Kojima and Carpenter 1997 and updated to 2006, Kojima et al. 2007).

### Ropalidia santoshae

Das & Gupta, 1989 rec. n.

http://species-id.net/wiki/Ropalidia_santoshae

Figure 2E

Ropalidia santoshae Das & Gupta, 1983: 422. Nomen nudum.
Ropalidia santoshae Das & Gupta, 1989: 111, 123, 156; Kojima et al. 2007: 382. Type locality: India.

#### Specimens examined.

INDIA: 1 ♀ (RMNH), Sikkim, Kambur, 3280 ft, 15.viii.1959, F. Schmid; 1 ♀ (RMNH), Assam, Div. Kamens, Bokhar, 2200–2500 ft, 28.v.1961, F Schmid (both paratypes labelled “*Ropalidia santoshae* Das L. l., det. J v d Vecht, 1981). CHINA: 1 ♀ (NWUM), Motuo, Tibet, 29°19'37.5"N, 95°19'44.6"E, 15.viii.2011, HL Yang & JB Wang; 10 ♀ (NWUM), with same locality data, but 10.vii.2013, T Li.

#### Remarks.

The specimens from China agree well with the examined paratypes from India.

#### Distribution.

China (Tibet); India. (Kojima and Carpenter 1997 and updated to 2006, Kojima et al. 2007).

### Ropalidia scitula

(Bingham, 1897) rec. n.

http://species-id.net/wiki/Ropalidia_scitula

Figure 2F

Icaria scitula Bingham, 1897: 387. Type locality: India (Sikkam).
Ropalidia scitula : van der Vecht 1941: 110, 142; Das and Gupta 1983: 428; Das and Gupta 1989: 113, 152, 172; Kojima et al. 2007: 389.

#### Specimens examined.

INDIA: 1 ♂ (RMNH), India, Kulara, Kumaon, 12000 ft, 4.viii.1958, F Schmid. MYANMAR: 1 ♀ (RMNH), Carin Chebá, Karen Hills, Kayin State, 900–1000 m, 5.viii.1988, L Fea. CHINA: 5 ♀ (ZJUH), Ruili, Yunnan, 1.v.1981, no. 812465, JH He.

#### Distribution.

China (Yunnan); India; Myanmar; Thailand. (Kojima and Carpenter 1997 and updated to 2006, Kojima et al. 2007).

### Ropalidia stigma

(Smith, 1858)

http://species-id.net/wiki/Ropalidia_stigma

Figure 2G

Polybia stigma Smith, 1858: 114. Type locality: Borneo (Sarawak).
Icaria stigma ; du Buysson 1913: 296.
Parapolybia stigma ; von Schulthess 1913: 164.
Ropalidia stigma stigma ; van der Vecht 1941: 110.
Ropalidia stigma nigrolineata van der Vecht, 1962: 18. Syn. by Nguyen et al. (2006).
Ropalidia stigma rufa van der Vecht, 1941: 130. Syn. by Nguyen et al. (2006).

#### Specimens examined.

CHINA: 1 ♀ (ZJUH), Guishan, Heyuan, Guangdong, 18.v.2002, no. 20028494, ZF Xu; 1 ♀ (NWUM), Luofu, Huizhou, Guangdong, 250 m, 12.vii.2004, CT Zhang; 1 ♀ (ZJUH), Dongzhong, Fangcheng, Guangxi, 8.xii.2001, ZQ He; 1 ♀ (ZJUH), Baise, Guangxi, 2.vi.1982, no. 822126, JH He; 9 ♀ + 2 ♂ (NWUM), Yaoqu, Mengla, Yunnan, 27–30.ix.2010, JL Tan; 7 ♀ + 2 ♂ (NWUM), Longmen, Shangyong, Yunnan, 26–27.ix.2010, JL Tan; 1 ♀ (GSFA) Xishuangbanna, Wangtianshu, Yunnan, 610 m, 23.iv. 2002, SP Sun; 1 ♀ (NWAM), Yaoqu, Mengla, Yunnan, 640 m, 6.v.1991, GC Liu & WZ Cai; 12 ♀ + 1 ♂ (NWUM), Beibeng, Motuo, Tibet, 10.viii.2011, HL Yang & JB Wang; 1 ♀ (NWUM), Motuo, Tibet, (29°19'37.5"N, 95°19'44.6"E), 15.viii.2011, HL Yang & JB Wang; 1 ♀ (ZJUH), Hongmao Village, Yuanmen, Baisha, Hainan, 29.iv.2010, JL Tan; 2 ♀ (ZJUH), Bawangling, Hainan, 19.viii.2000, no. 200104356, -67, ZF Xu; 1 ♀ (CATAS), Leguang Farm, Ledong, Hainan, 14.vii.2011, 18°37'0"N, 109°6'39"E, no. 07018, WJ Zhu; 4 ♀ + 2 ♂ (CATAS), Nada, Danzhou, Hainan, 19°05'17"N, 109°34'50"E, 143 m. INDONESIA: 2 ♀ + 2 ♂ (RMNH), Sumatra/Ind., viii-ix.1989, F. Moussault. VIETNAM: 5 ♀ (RMNH), S. Vietnam: Dak Lak, Chu Yang Sin N.P., Krong K’Mar, 550–610 m, 21–26.x.2005, C van Achterberg & R de Vries, (mesoscutum with a pair of narrow yellow stripes vaguely visible in the reddish brown mark); 1 ♀ (RMNH), S. Vietnam: Dông Nai, Cát Tien N.P., *Ficus* trail, Mal. traps, c 100 m, 9–30.iv.2007, MP Quy & NT Manh; 1 ♀ (RMNH), same data, but Botanical garden, Mal. traps 14–19, c 100 m, 14–20.v.2007, C van Achterberg & R de Vries; 1 ♀ (RMNH), same data, but c 100 m, 6.x.2005, Ecotrail, C van Achterberg & R de Vries (similar to Chinese specimens with small yellow spots on second tergite and sternite). MALAYSIA: 1 ♀ (RMNH), Penang Isl., Malaya, Batu Feringgi, 25.ii.1963, MA Lieftinck; 2 ♀ (RMNH), Perlis, Bukit Bingtang, Forest Res. (Kangar), 23.ii.1963, MA Lieftinck.

#### Remarks.

Specimens from Tibet are slightly darker than those from Yunnan and the basal yellow marks of the second sternite are very small or lacking. Chinese specimens have the gena slightly wider and the first tergite longer than other specimens, 3.0–3.5 times as long as high with small yellow patches. Specimens from Vietnam and Malaysia have the first tergite shorter, about 2.9 times as long as high with large yellow patches.

#### Distribution.

China (Hainan, Guangdong, Guangxi, Yunnan, Tibet); Bali; Borneo; India; Java; Malay Peninsula; Myanmar; Nepal; Philippine Islands; Sri Lanka; Sumatra; Thailand; Vietnam. (Kojima and Carpenter 1997 and updated to 2006, Nguyen et al. 2006, Kojima et al. 2007).

### Ropalidia sumatrae

(Weber, 1801)

http://species-id.net/wiki/Ropalidia_sumatrae

Figure 2H

Vespa sumatrae Weber, 1801: 103. Type locality: Sumatra.
Icaria sumatrae ; de Saussure 1854: 241.
Ropalidia sumatrae sumatrae van der Vecht, 1962: 35.
Ropalidia sumatrae lugubris van der Vecht, 1941: 104. Syn. by Kojima et al. (2007).
Icaria speciosa de Saussure, 1855: 374. Type locality: Sumatra. Syn. by van der Vecht (1941).

#### Specimens examined.

CHINA: 1 ♀ (NWAM), Menglun, Yunnan,19.v.1991, YL Wang & RG Tian; 1 ♀ (ZJUH), id., but 12.iv.1981, JH He, no. 811796; 2 ♀ (ZJUH), Sanchahe, Yunnan, 10.iv.1981, JH He, no. 811812; 1 ♀ (ZJUH), Mt. Youle, Yunnan,17.iv.1981, JH He, no. 811873. MALAYSIA: 2 ♂ (RMNH), Sabah, Brumas, NBT, 23–27.i.1973, K.M. Guichard; 1 ♂ (RMNH), Borneo, Sarawak, trail Bario-Pa Lungan, 3°48'N, 115°34'E, 1100 m, 23.ii.1987, J Huisman; 1 ♀ (RMNH), SW. Sabah, near Long Pa Sia (West), c. 1200 m, 2–14.iv.1987, Mal. trap 7, C van Achterberg. INDONESIA: 1 ♀ (RMNH), N. Sumatra: Allas Valley, near Gumpang, 13.vi.1972, 3°48'N, 97°29'E, J Krikken; 2 ♀ (RMNH), N. Sumatra, Aceh, Ketamba, 400 m, iv.1995, Mal. trap, near edge rainforest, Y van Nierop & C van Achterberg.

#### Remarks.

Lee (1982, 1985) used *Ropalidia speciosa* still as a valid species.

#### Distribution.

China (Yunnan); Bangka; Borneo; India; Malaysia (Sabah, Sarawak) (rec. n.); Myanmar; Sumatra; Thailand; Vietnam. (Lee 1982, 1985, Kojima and Carpenter 1997 and updated to 2006, Kojima et al. 2007).

### Ropalidia taiwana

Sonan, 1935

http://species-id.net/wiki/Ropalidia_taiwana

Figure 2I

Ropalidia taiwana Sonan, 1935: 199. Type locality: China (Taiwan: Shinchiku).
Ropalidia taiwana taiwana ; Iwata 1976: 295; Lee 1982: 83, 92; 1985: 46, 52.
Ropalidia taiwana var. *koshunensis* Sonan, 1935: 199, type locality: China (Taiwan: Koshun). Syn. by Starr (1992).
Ropalidia taiwana koshunensis ; Iwata 1969: 367.
Ropalidia formosana Kuo, in Kuo and Yeh 1987: 84, type locality: China (Taiwan: Wufeng). Syn. by Starr (1992).

#### Specimens examined.

CHINA: Type material of *Ropalidia taiwana*, no. 90-96 in TARI: 1 ♀, Holotype, Shinchiku, Formosa 18.vii.1-30, J Sonan, K Miyake; paratypes: 1 ♀, id.; 1 ♀, Hassenzan, 23.x.1932, K Nomura; 1 ♂, Horisha, 2.xii.1916, T Shiraki; 1 ♀, Koshun, Formosa, 1918, iv.25–v.25. J Sonan, K Miyake, M Yoshino; 1 ♀, Urai, vii.1931, J Sonan; 1 ♀ (RMNH), Eassenzan, Formosa, 23.vi.1934, L Gressitt. Type material of *Ropalidia taiwana* var. *koshunensis* Sonan, 1935, confirmed by J Kojima & F Saito, 2010: 1 ♀, Holotype, Kuraru, 13.x.1926, J Sonan, “*Ropalidia taiwana Sonan var. formosensis* Sonan, DET. J SONAN. Type label, “97”; Paratypes: 6 ♀ + 5 ♂ with same data; 2 ♀, with same data but 14.x.1926; 1 ♀ “Koshun 1918.iv.25–v.25, J Sonan, K Miyake, M Yoshino; 1 ♂, Koushun, 25.iii.1930, T Shiraki”. 1 ♀ (RMNH), Rokki, Formosa, 20.v.1934, L Gressitt; 2 ♀ (RMNH), Kosempo, Formosa, 11.vii.1911, H Sauter; 2 ♀ (RMNH), Taihorin, Formosa, 10.v, H Sauter; 1 ♂ (RMNH), Taihorinsho, Formosa, H Sauter; 1 ♀ (RMNH), Ku-ling, Jiangxi, 13.ii.1935, O Piel; 1 ♀ (RMNH), Kuatun, Fujian, 27°40'N, 117°40'E, 2300 m, 14.iv.1938, LJ Klapperich; 1 ♀ (ZJUH), Mt. Baxian, Heiping, Taizhong, Taiwan, 24°11'N, 121°E, 4–5.vi.2011, P Tang. MYANMAR: 2 ♀ (RMNH), Sadon, 1200 m, 28.vi.–5.vii.1934, R Malaise; 2 ♀ (RMNH), Taunggyi, S. Shan States, 1.viii.–22.ix.1934, R Malaise.

#### Distribution.

China (Jiangxi, Fujian, Taiwan); Myanmar. (Kojima and Carpenter 1997 and updated to 2006)

### Ropalidia variegata

(Smith, 1852)

http://species-id.net/wiki/Ropalidia_variegata

Figure 2J

Epipona variegata Smith, 1852: 48. Type locality: India (Poona).
Icaria variegata ; de Saussure 1854: 237.
Ropalidia variegata ; Bequaert 1918: 247; van der Vecht 1941: 104; Kojima et al. 2007: 387.
Ropalidia variegata variegata ; van der Vecht 1941: 112.
Icaria pendula Smith, 1857: 98. Type locality: India (Barelly). Syn. by van der Vecht (1941).
Ropalidia pendula ; Richards 1978: 58.

#### Specimens examined.

INDIA: 1 ♀, Type, 60-15, E.I.C., with a handwritten label “*variegata* Type, Sm.”, Capt. F. Downes, BM Type 18.846, van der Vecht (1941) recorded it is Holotype, locality: Poona, India; 1 ♂, India, with a label: “*Ropalidia pendula* Smith” (BMNH); 1 ♀ (RMNH), India, Pinjore, (identified as *Ropalidia variegata* by Dover, 1921). PAKISTAN: 1 ♀ (RMNH), Karachi, 25.i.1934, R Malaise.

#### Remarks.

No specimens from China were available, but the species is included in the key because it has been listed for China. Liu (1936–37) listed *Ropalidia variegata* (Smith) from China in his catalogue and *Ropalidia variegata variegata* (Smith) was reported from China by Lee (1982, 1985). However, considering that Lee (1982, 1985)’s description of the male antenna with its hooked terminal segment, we suspect misidentification of *Ropalidia variegata*.

#### Distribution.

China; India; Malay Peninsula; Myanmar; Nepal; Pakistan; Sulawesi. (Kojima and Carpenter 1997 and updated to 2006).

## Supplementary Material

XML Treatment for Ropalidia artifex

XML Treatment for Ropalidia bicolorata

XML Treatment for Ropalidia binghami

XML Treatment for Ropalidia birmanica

XML Treatment for Ropalidia cyathiformis

XML Treatment for Ropalidia fasciata

XML Treatment for Ropalidia flavopicta

XML Treatment for Ropalidia hongkongensis

XML Treatment for Ropalidia malaisei

XML Treatment for Ropalidia marginata

XML Treatment for Ropalidia mathematica

XML Treatment for Ropalidia obscura

XML Treatment for Ropalidia opifex

XML Treatment for Ropalidia ornaticeps

XML Treatment for Ropalidia parartifex

XML Treatment for Ropalidia rufocollaris

XML Treatment for Ropalidia santoshae

XML Treatment for Ropalidia scitula

XML Treatment for Ropalidia stigma

XML Treatment for Ropalidia sumatrae

XML Treatment for Ropalidia taiwana

XML Treatment for Ropalidia variegata
